# The mosquito *Aedes aegypti* requires a gut microbiota for normal fecundity, longevity and vector competence

**DOI:** 10.1038/s42003-023-05545-z

**Published:** 2023-11-13

**Authors:** Ruby E. Harrison, Xiushuai Yang, Jai Hoon Eum, Vincent G. Martinson, Xiaoyi Dou, Luca Valzania, Yin Wang, Bret M. Boyd, Mark R. Brown, Michael R. Strand

**Affiliations:** 1 Department of Entomology and Center for Tropical and Emerging Global Diseases, University of Georgia, Athens, GA 30602 USA; 2 Department of Biology, University of New Mexico, Albuquerque, NM 87131 USA; 3 Institut Curie, 20 Rue d’Ulm, 75238, Paris, Cedex 05 France; 4 Center for Biological Data Science, Virginia Commonwealth University, Richmond, VA 23284 USA; 5 Present Address: Department of Cellular Biology and Center for Tropical and Emerging Global Diseases, University of Georgia, Athens, GA 30602 USA

**Keywords:** Entomology, Microbial ecology

## Abstract

Mosquitoes shift from detritus-feeding larvae to blood-feeding adults that can vector pathogens to humans and other vertebrates. The sugar and blood meals adults consume are rich in carbohydrates and protein but are deficient in other nutrients including B vitamins. Facultatively hematophagous insects like mosquitoes have been hypothesized to avoid B vitamin deficiencies by carryover of resources from the larval stage. However, prior experimental studies have also used adults with a gut microbiota that could provision B vitamins. Here, we used *Aedes aegypti*, which is the primary vector of dengue virus (DENV), to ask if carryover effects enable normal function in adults with no microbiota. We show that adults with no gut microbiota produce fewer eggs, live longer with lower metabolic rates, and exhibit reduced DENV vector competence but are rescued by provisioning B vitamins or recolonizing the gut with B vitamin autotrophs. We conclude carryover effects do not enable normal function.

## Abstract (web summary)

A study of the mosquito *Aedes aegypti* indicates normal adult longevity, reproduction and vector competence to transmit dengue virus depends on having a gut microbiota that can provision B vitamins.

## Introduction

Dietary deficiencies cause a variety of defects in animals including arrested development^1^. Most animals also host a gut microbiota that can be affected by diet or influence how animals respond to changes in diet^2,3^. Wide-ranging feeding habits make insects valuable subjects for understanding how diet and gut microbes interact^4^. Holometabolous insects that undergo complete metamorphosis are also of interest because larvae and adults often consume different diets^5^. Metamorphosis is thought to be a key factor in the diversification of insects into the largest animal group on Earth^6^, but how gut microbes affect responses to dietary changes between life stages remains unclear^7^.

Mosquitoes are holometabolous insects that include many vector species which vector blood-borne pathogens to humans or other vertebrates^8^. All mosquitoes are aquatic during their immature stages with larvae usually feeding as detritivores before pupating. Terrestrial adults of both sexes consume water and sugar sources, while females usually must also blood feed on a host to produce eggs^8–10^. Each gonadotrophic cycle is activated by a separate blood meal while successive blood meals underly how pathogens are acquired and transmitted between hosts^8–10^. Bacteria and other microbes continuously move between the larval gut and aquatic environment, while adults maintain a gut microbiota that includes species carried over from the larval stage and that are acquired by imbibing water, sugar feeding, and mating^11–13^.

Insects that feed on nutrient-deficient diets often host heritable microbes that function as nutritional symbionts^4^, but no heritable microbes with functions in nutrient provisioning are known in mosquitoes^11^. Larvae can experience resource limitations in the field but in the laboratory are fed nutrient-rich diets that are thought to provide all required resources^14,15^. Nonetheless, several mosquito species in axenic (AX) cultures with no commensal microbes arrest as first instars when fed laboratory diets under standard conditions (temperatures of 25–28 °C and 12–16 h light: 8–12 h dark photoperiods) that mimic what larvae usually experience in the field^16–18^. Arrest is also associated with alterations in metabolism, gut function, and nutrient storage^19–21^. Many species of bacteria have been identified in mosquito larvae and adults that are reared under non-sterile conditions which we hereafter refer to as conventional (CN) larvae or adults^11^. Several species of bacteria including *Escherichia coli* K12 MG1655 rescue larval development to adulthood when added alone or as defined communities to AX cultures, creating a gnotobiotic (GN) culture, but these microbes have no rescue effects if dead^16–24^. No larval rearing diets have been identified that enable larvae in AX cultures to develop into adults if maintained under standard rearing conditions, whereas some diets have been identified that support development of AX larvae into adults if maintained in darkness^25–27^. These findings thus collectively suggest larval rearing diets lack or lose factors under standard conditions that mosquito larvae require for development, while some species of living bacteria can provision them.

Like other insects, mosquitoes cannot synthesize essential nutrients including B vitamins that must be acquired from other sources^26,28^. To identify the factors lost from undefined diets under standard rearing conditions, we recently developed a defined (holidic) medium named H + LA that contains all essential nutrients mosquitoes are thought to require plus lactalbumin^26^. H + LA supports development of AX *A. aegypti* larvae into adults in darkness but first instars arrest under standard conditions as occurs with undefined diets due to photosensitivity of select B vitamins^26^. Riboflavin is highly photosensitive, decaying in 6–12 h under standard conditions, and is the only nutrient that causes larvae to arrest as first instars if omitted from H + LA^26^. AX larvae likewise arrest as first instars if maintained with an *E. coli* K12 riboflavin auxotroph but develop normally into adults if maintained with wild-type *E. coli* that can synthesize riboflavin and all of the other B vitamins in H + LA^26^. AX larvae molt to second or third instars before arresting when fed H + LA lacking thymidine or pyridoxine, while the proportion of larvae that pupate and emerge as adults is reduced in the absence of folate^26^. Omission of the other B vitamins in H + LA (pantothenate, biotin, nicotinate) does not significantly reduce development into adults^26^. These findings indicate larvae require bacteria like *E. coli* which can provision B vitamins that are absent or lost from dietary sources under standard rearing conditions. Other studies of *A. aegypti* have also shown that folate deficiency reduces the proportion of larvae that pupate and eclose as adults^18^, while studies of other insects indicate deficiencies in thiamin and other B vitamins adversely affect development or cause long-term (intergenerational) defects^28–30^.

The sugar and blood meals adult mosquitoes consume are rich in carbohydrates or protein respectively but are deficient for other nutrients including B vitamins^31,32^. Obligately hematophagous arthropods like ticks and lice host intracellular symbionts that are thought to fully or partially compensate for nutrient deficiencies in blood^32^. In contrast, facultatively hematophagous insects like mosquitoes have been suggested to avoid nutrient deficiencies by relying on resources larvae consume and carry over to the adult stage^32^. However, studies focusing on mammals indicate B vitamins are not stored and require daily repletion^33^, which for holometabolous insects suggests carryover from the larval stage may be insufficient to provision adults. Prior studies of carryover effects in mosquitoes have all used CN adults or adults that developed from GN larvae but were maintained under non-sterile conditions which enables acquisition of other microbes by feeding^17,34–38^. The presence of a gut microbiota in adults could thus provision B vitamins and other resources while also obscuring whether carryover effects from the larval stage enable normal function. Some studies have used short-term treatment of adults with antibiotics targeting bacteria to reduce the microbiota in CN adults^39,40^. Other studies though indicate resistant bacteria and non-susceptible microbes like fungi are present in larvae or adults, while also indicating short-term treatment with antibiotics does not produce AX mosquitoes^41,42^. Alternatively, approaches have been developed for producing AX adults by rearing GN larvae with particular strains of *E. coli* that are cleared by antibiotics or other methods^16,18^. Results described above also indicate AX adults can be produced by axenic rearing in darkness^26,27^. Here we asked if carryover effects from the larval stage enable AX adults to function like CN adults by measuring egg production, longevity and competence to vector dengue virus (DENV). We report that adults require a gut microbiota that includes B vitamin autotrophs for normal function.

## Results

### AX adult females lay smaller egg clutches than CN females

Because prior studies of carryover effects from larvae to adults have used non-sterile adults with a gut microbiota, we included CN adults as a treatment but also reared AX and CN adults as similarly as possible to minimize differences when making comparisons between treatments. Toward this end, we earlier reported that AX *A. aegypti* larvae produced from surface sterilized eggs do not develop beyond the first instar when maintained under standard rearing conditions but develop normally into adults in GN cultures containing certain species of bacteria in our laboratory culture or certain species of bacteria from field-collected *A. aegypti* including *E. coli* which is a known B vitamin autotroph^16,26,41^. *E. coli* K12 MG1655 is highly susceptible to ampicillin^20,26^. Treating GN cultures containing *E. coli* K12 MG1655 with ampicillin as second or third instars results in larvae molting to third or fourth instars but then cease to further grow because clearance of this bacterium results in riboflavin deficiency that causes developmental arrest^26^. In this study, we added *E. coli* K12 MG1655 to AX cultures containing first instars fed sterile rat chow mix (RCM) diet we use for general rearing but added ampicillin when larvae were second day fourth instars (Supplementary Fig. S1). This resulted in almost all individuals molting to the pupal stage that was briefly surface sterilized with bleach before transfer to sterile water from which AX adults eclosed (Supplementary Fig. S1). The AX status of these adults was confirmed by no detection of bacterial or fungal DNA by PCR or microbial colonies when homogenates from individual adults were cultured on Luria broth (LB) plates (Supplementary Fig. S2a). Development time to pupation, female size as estimated by wing length, and sex ratio did not differ when compared to CN adults, or adults from GN cultures containing *E. coli* that were not treated with ampicillin (Supplementary Fig. S2b). The second approach we used to produce AX adults consisted of feeding AX larvae from surface sterilized eggs H + LA in 6-well culture plates in darkness (Supplementary Fig. S1). These larvae take 1-2 days longer to pupate but adult females do not differ in size from CN adults^26^. CN adults used in experiments were also reared in 6-well culture plates from AX larvae that hatched from sterile eggs and were fed sterile RCM diet. However, larvae were inoculated with a mixed community of microbes prepared from our general *A. aegypti* culture, which earlier studies showed to result in adults having a mixed community of gut microbes that were carried over from the larval stage by transstadial transmission^16^. Although unnecessary for normal development, GN larvae with *E. coli* and CN larvae were also reared in darkness for consistency with AX larvae reared without microbes. Resulting adults from each treatment were also maintained under identical physical conditions (Supplementary Fig. S1).

**Fig. 1:**
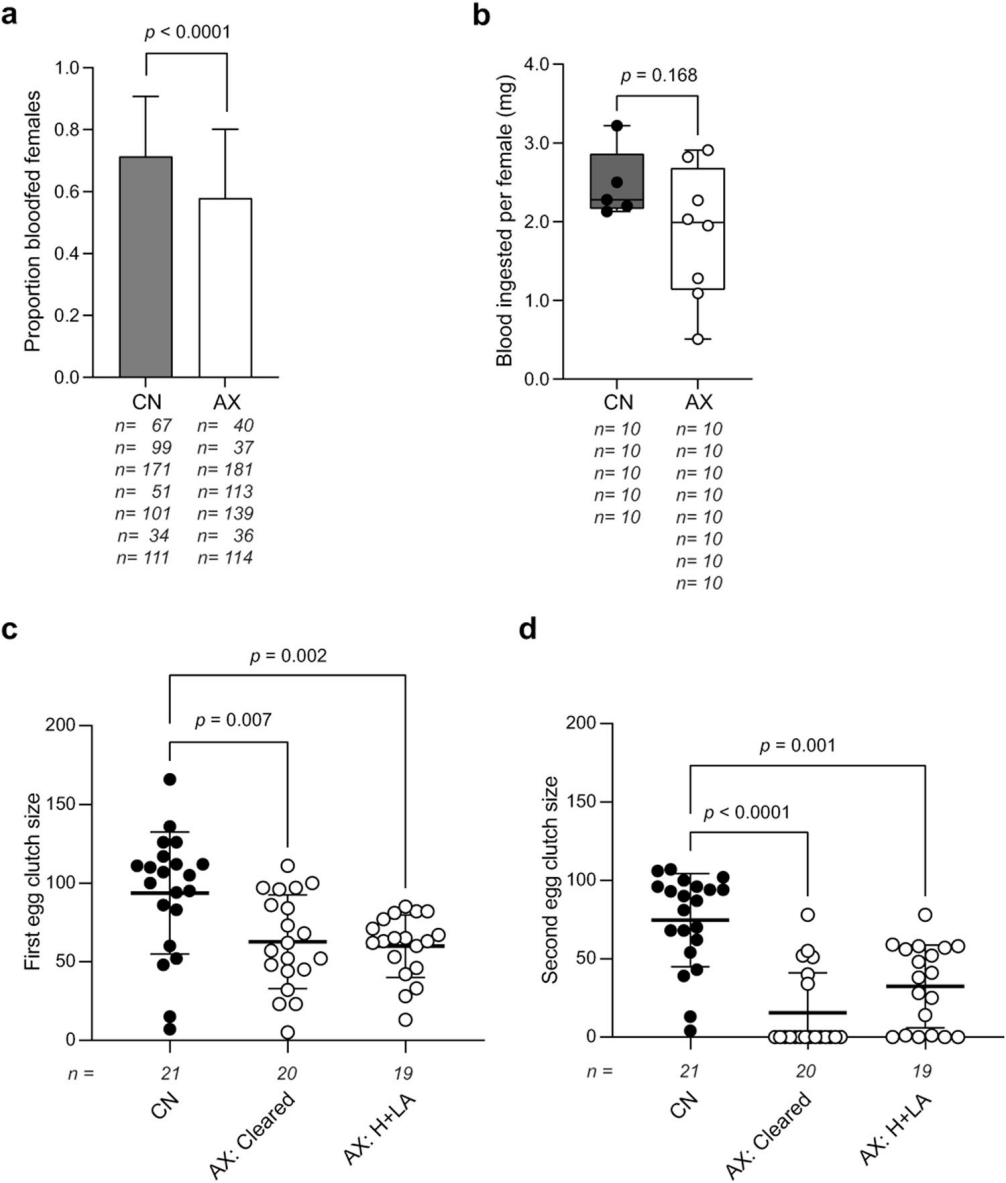
AX females lay fewer eggs than CN females. **a** Proportion of CN females and AX females produced by clearance of *E. coli* K12 that blood fed when provided a blood meal. **b** Box-plots showing the mass (mg) of blood meals ingested by CN and AX females produced by clearance. **c** Egg clutch sizes laid by CN females, AX females produced by clearance (AX: Cleared) and AX females produced axenic rearing (AX: H + LA) after a first blood meal. **d** Egg clutch sizes laid by CN females, AX females produced by clearance and AX females produced axenic rearing after a second blood meal. In **a**, data show the proportion of females that blood fed ±95% confidence intervals in seven independent replicates. Exact numbers of CN and AX females per replicate are indicated below the x axis. Statistical significance was determined by contingency table analysis. In **b**, box plots show the median (center line), upper and lower quartiles (outer lines in boxes) and minimum, and maximum values (whiskers) determined by weighing 5 independent replicates of 10 pooled CN females or 8 independent replicates of 10 pooled AX females before and after blood feeding. Statistical significance was determined after assessment of homogeneity of variances by an unpaired Student’s *t* test. In **c** and **d**, data show the mean ± SD number of eggs laid by females with exact number of individuals measured shown below the x axis for each treatment. Statistical significance was determined after assessment of homogeneity of variances by Kruskal–Wallis and post-hoc Dunn’s tests. Exact *p* values are indicated in each panel of the figure.

Developmental arrest of AX larvae is associated with multiple defects including reduced production of ATP due to deficiencies in riboflavin-derived cofactors required for electron transport^19–21,26^. Prior results additionally suggested that similar to mammals^33^, *A. aegypti* does not store riboflavin which underlies why GN second and third instars arrest after molting to the third or fourth instars respectively when a complete B vitamin autotroph like *E. coli* is cleared^26^. Since blood meal digestion and egg production are both metabolically costly^9,43^, we first asked if AX and CN adult females exhibited differences in blood feeding and egg production. Using AX adult females produced by clearance of *E. coli* K12 showed that a smaller proportion blood fed when compared to CN females (Fig. 1a), but the females that did feed consumed equivalent amounts of blood as CN females (Fig. 1b). In contrast, AX females produced by clearance or axenic rearing by feeding larvae H + LA diet laid smaller egg clutches than CN females (Fig. 1c). We also assessed the number of eggs laid in a second gonadotrophic cycle because carryover of macronutrients required for egg development have previously been shown to primarily contribute to production of a first clutch^9,44^. Again, AX females produced by clearance or axenic rearing laid smaller second clutches than CN females (Fig. 1d). Dissection of spermathecae from ten randomly selected AX females produced by clearance or AX rearing of larvae showed that all were mated indicating the smaller clutch sizes were not due to being unfertilized. We also measured the proportion of eggs that hatched for 22 egg clutches (1462 eggs total) laid by AX females produced by clearance and 27 egg clutches (1,555 eggs total) laid by CN females after the first blood meal was consumed. No difference in hatch rates was detected (66% for AX females, 65% for CN females, *p* = 0.4, contingency table analysis), but we did not rear these larvae to assess whether differences existed in subsequent growth, pupation or adult emergence.

### Axenic females exhibit defects in egg maturation and nutrient storage

**Fig. 2:**
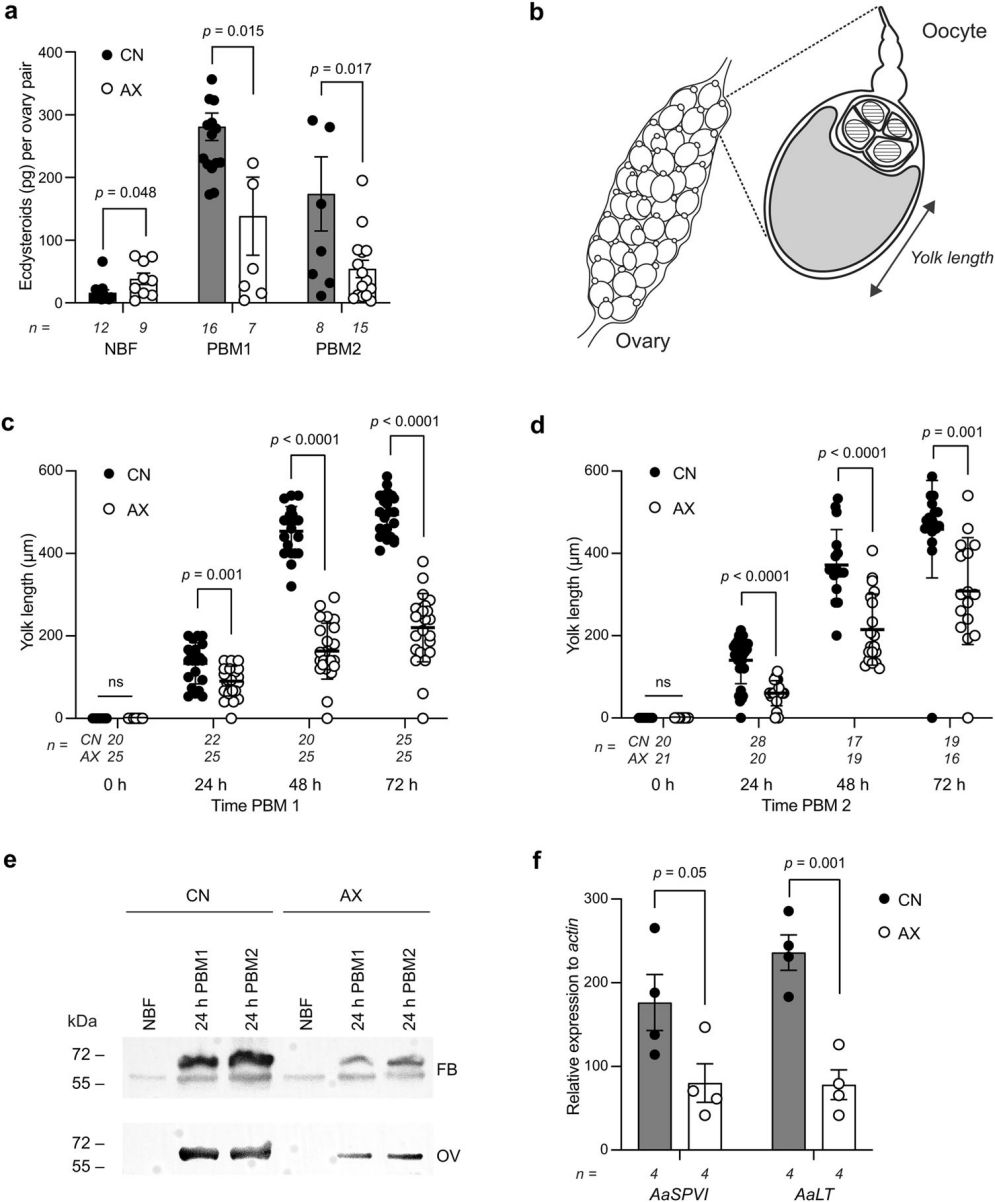
AX females exhibit multiple defects during oogenesis. **a** Ecdysteroids produced per ovary pair by CN females and AX females produced by clearance. Samples were collected from 4 day old non-blood fed females (NBF), 20 h post-blood meal (PBM) for the first gonadotrophic cycle (PBM1), and 20 h post-blood meal for the second gonadotrophic cycle (PBM2). **b** Diagram of an ovary and an individual oocyte indicating how yolk deposition per oocyte is estimated by yolk length (μm). Yolk packaged into the oocyte is indicated in gray while nurse cells (trophocytes) are located distally. **c**, **d** Yolk length in oocytes from CN and AX females produced by clearance at 4 days post-emergence before blood feeding (NBF), 72 h PBM for the first gonadotrophic cycle (PBM1), and 72 h PBM for the second gonadotrophic cycle (PBM2). **e** Immunoblots showing the small subunit(s) of vitellogenin in the fat body (FB) (upper) and ovaries (OV) (lower) of CN and AX females produced by clearance. Samples were collected from 4-day old NBF females, 24 h PBM1 females, and 24 h PBM2 females. **f** Mean relative transcript abundance for serine protease VI (*AaSPVI*) and late-phase serine protease (*AaLT*) in the midguts of CN and AX females produced by clearance at 24 h of PBM1. In **a**, **c** and **d**, data show mean amounts ±SD with number of independent replicates per treatment shown below the x axis of each graph with each replicate for ecdysteroids determined from 1 ovary pair from two females and each replicate for yolk length determined by taking the average from measuring 3 oocytes from individual females. Statistical significance was determined after assessment of homogeneity of variances by unpaired Student’s *t* tests. In **e**, treatments were normalized by loading each lane with tissue equivalents: 1/5 fat body per lane (top) and 1/10 ovary per lane (bottom). Size markers in kilodaltons (kDa) are shown to the left of each blot. In **f**, data show mean expression ±SE with data normalized to a reference gene (*actin*). Number of independent replicates per treatment (2 midguts per replicate) are indicated below the x axis. Statistical significance was determined by unpaired Student’s *t* tests. Exact *p* values are indicated in each panel of the figure.

Each gonadotrophic cycle in *A. aegypti* consists of a previtellogenic phase before blood feeding that produces primary follicles followed by a vitellogenic phase after blood feeding that stimulates primary follicles to develop into mature eggs^43^. Key events during the vitellogenic phase include: 1) release of neuropeptide hormones in response to blood feeding that activate primary follicles and ecdysteroid production by the ovaries; 2) biosynthesis of the yolk protein vitellogenin by the fat body in response to ecdysteroids, insulin-like peptides (ILPs) and target of rapamycin signaling, 3) yolk packaging into oocytes of primary follicles, and 4) digestion of the blood meal, which primarily consists of protein, by midgut-produced serine-like protease VI (AaSPVI) and late trypsin (AaLT) that ecdysteroids and ILPs co-regulate^45–48^. Since AX females produced by clearance of *E. coli* or axenic rearing both laid fewer eggs than CN females, we used the former to determine if smaller clutches were associated with defects in egg formation or oviposition. Results showed that the ovaries produced less ecdysteroids while primary follicles packaged less yolk, which is a well-established measure of egg maturation (Fig. 2a–d)^45,49^. The fat body also produced less vitellogenin in AX than CN females during both gonadotrophic cycles (Fig. 2e), while focusing on the first gonadotrophic cycle indicated transcript abundances for *AaSPVI* and *AaLT* in the midgut were lower in AX than CN females (Fig. 2f).

**Fig. 3:**
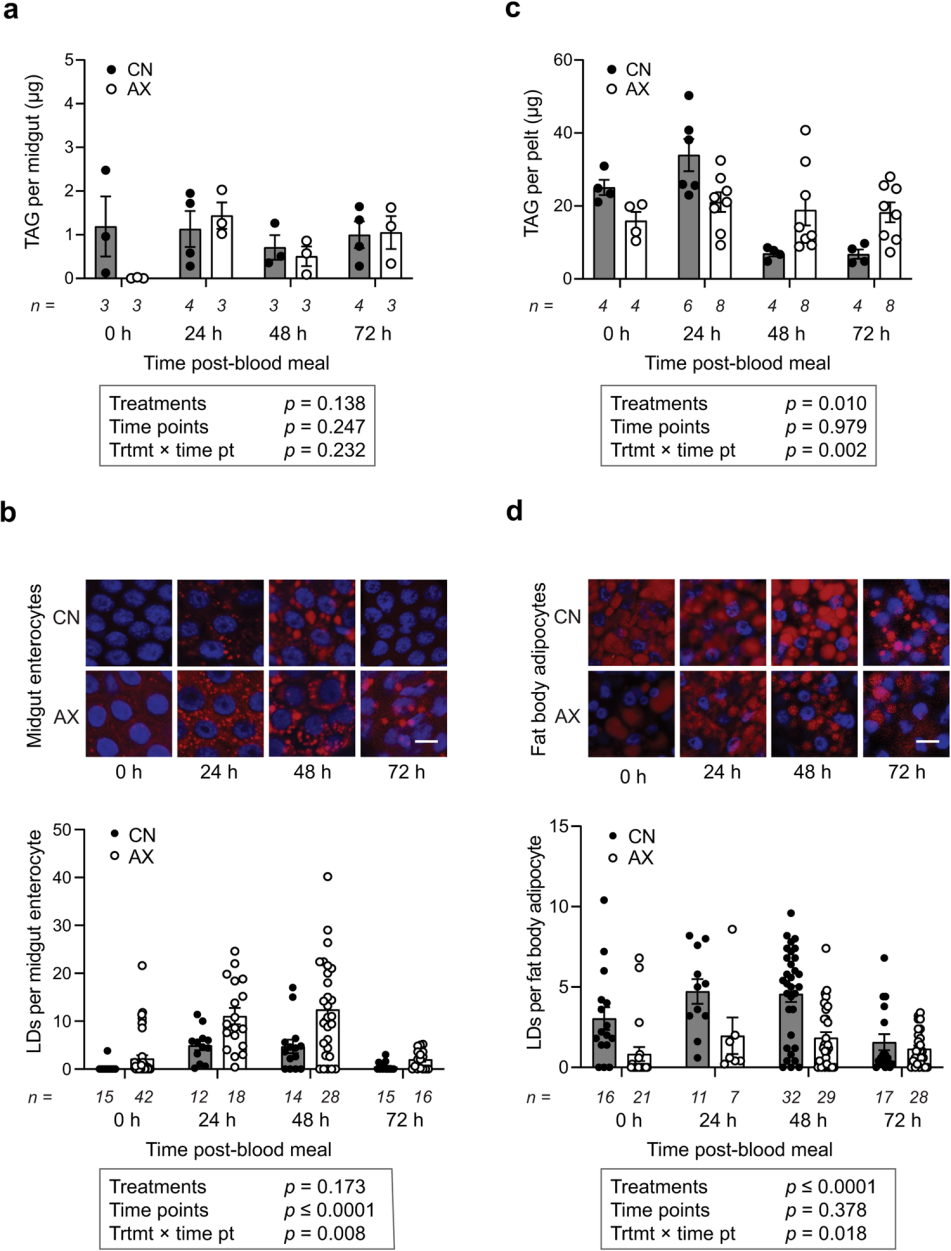
AX females store and mobilize less lipid in the fat body during a gonadotrophic cycle. **a** TAG stores in the midguts of CN and AX females produced by clearance at 0, 24, 48 and 72 h PBM of the first gonadotrophic cycle. **b** Confocal microscopy images of lipid droplets (red) in midgut enterocytes (upper panel) and number of lipid droplets (LDs) per enterocyte (lower panel) in CN and AX females produced by clearance at 0, 24, 48 and 72 h PBM of the first gonadotrophic cycle. **c** TAG stores in the fat bodies of CN and AX females produced by clearance at 0, 24, 48 and 72 h PBM of the first gonadotrophic cycle. **d** Confocal microscopy images of LDs (red) in fat body adipocytes (upper panel) and number of LDs per adipocyte (lower panel) in CN and AX females produced by clearance at 0, 24, 48 and 72 h PBM of the first gonadotrophic cycle. In **a** and **c**, data show mean TAG ± SE per midgut or abdominal pelt containing the fat body with number of independent replicates per treatment (2 midguts or pelts per replicate) and time point shown below the x axis of each graph. In **b** and **d** (upper panel), LDs are stained with Nile red (red) and nuclei are stained with Hoechst 33342 (blue). Scale bar in the lower right micrograph in **b** equals 20 μm with all other images at the same magnification, while scale bar in the lower right micrograph in **d** equals 10 μm with all other images at the same magnification. In **b** and **d** (lower panel), data show mean ± SE with number of independent replicates (individual midguts or pelts with LDs measured in 5 cells to generate an average number of LDs per cell per female) shown below the x axis for each treatment and time point. Statistical significance was determined with linear repeated measures mixed effects models to incorporate the effects of treatment, time point, and the interaction between these factors. Exact *p* values are indicated in each panel of the figure.

Macronutrients carried over from the larval stage are often referred to as teneral reserves that adults primarily store as neutral lipid droplets (LDs) in the fat body^9,50,51^. Adult females additionally convert carbohydrates from sugar feeding and protein from blood feeding to triacylglycerol (TAG) and other neutral lipids in the midgut that are transported and stored in the fat body as LDs^50,51^. We detected only low levels of TAG in the midguts of CN and AX adults produced by clearance after blood feeding, which was consistent with earlier results showing that the mosquito midgut rapidly transports neutral lipids to the fat body (Fig. 3a)^21,50,51^. LDs transiently increased more in the midguts of AX than CN females after blood feeding but declined to similarly low levels by 72 h PBM (Fig. 3b). In contrast, lipid stores as measured by TAG and LDs in the fat body were larger in 4 day old non-blood fed (=0 h PBM) CN than AX females (Fig. 3c, d). TAG and LDs per adipocyte also declined in CN females after blood feeding which was consistent with nutrient mobilization that normally occurs^51^, whereas TAG and LDs did not decline after AX females blood fed (Fig. 3c, d). Thus, the smaller clutch sizes laid by AX females reflected: 1) lower ecdysteroid synthesis, 2) lower vitellogenin production and yolk deposition into primary follicles, and 3) reduced lipid stores in the fat body.

### AX females provisioned with B vitamins or B vitamin autotrophs lay similar clutch sizes as CN females

**Fig. 4:**
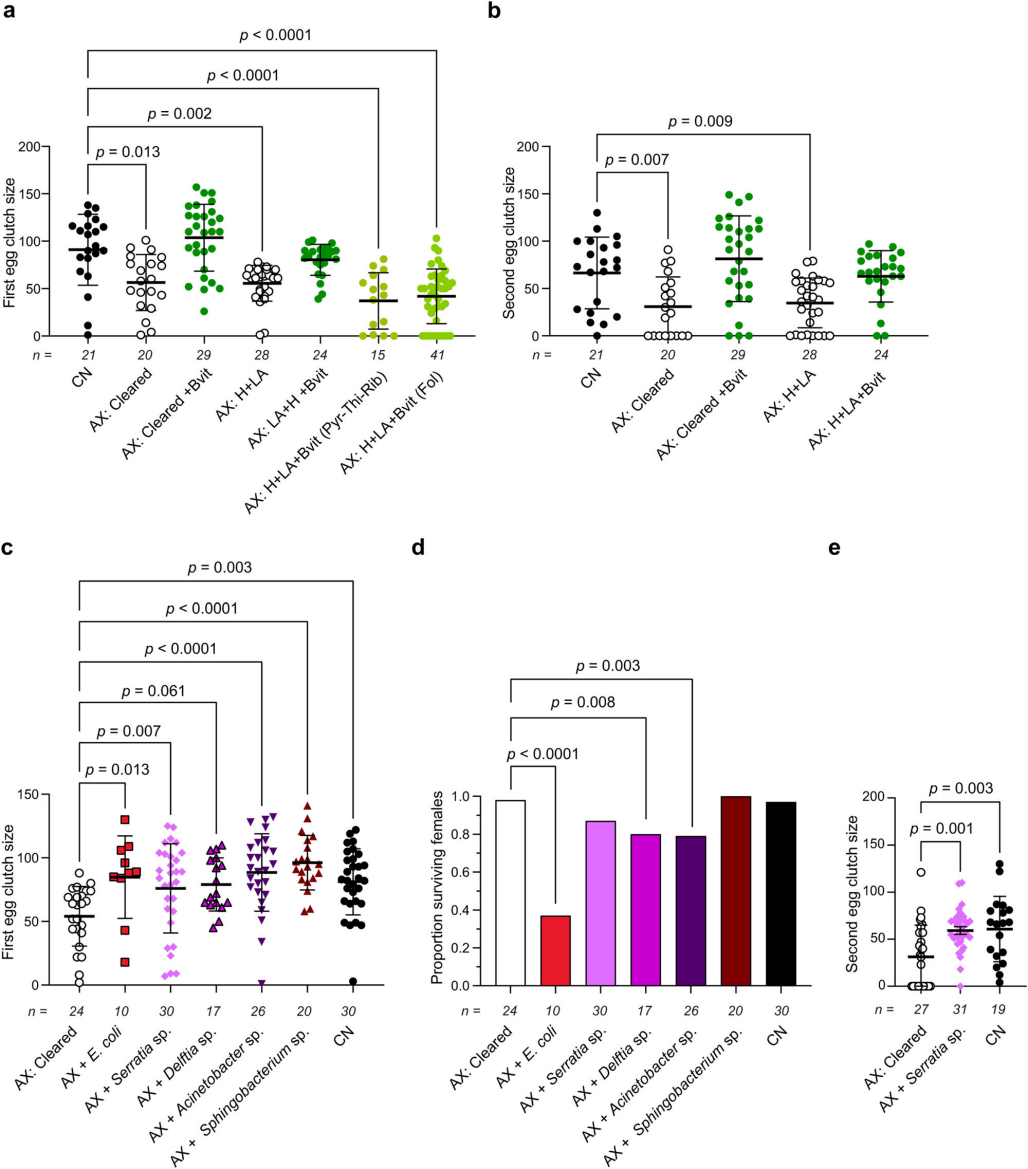
B vitamins and B vitamin autotrophs increase clutch sizes laid by AX females to similar levels as CN females. **a** Clutch sizes laid after the first gonadotrophic cycle by CN females, AX females produced by clearance of *E. coli* (AX: Cleared), or AX females produced by axenic rearing (AX: H + LA). AX treatments were also provisioned with all of the B vitamins in H + LA (+Bvit), only pyridoxine, thiamin and riboflavin (AX: H + LA + Bvit (Pyr-Thi-Rib)), or only folate (AX: H + LA + Bvit (Fol)). **b** Clutch sizes laid after the second gonadotrophic cycle by CN females, AX females produced by clearance, or AX females produced by axenic rearing. AX treatments were also provisioned with all B vitamins. **c** Clutch sizes laid after the first gonadotrophic cycle by AX females produced by clearance, AX females produced by clearance that were inoculated as adults with *Serratia* sp.*, Delftia* sp.*, Acinetobacter* sp., or *Sphingobacterium* sp., or CN females. **d** Proportion of females alive 24 h after laying eggs for the treatments shown in **c**. **e** Clutch sizes laid after the second gonadotrophic cycle by AX females produced by clearance, AX females produced by clearance that were inoculated as adults with *Serratia* sp., or CN adults. In **a**–**c** and **e**, data show mean ± SD with exact number of individuals analyzed per treatment shown below the x axis of each graph. Statistical significance was determined after assessment of homogeneity of variances followed by Kruskal–Wallis and post-hoc Dunn’s tests (**a**–**c**) or one-way ANOVA and a post-hoc Dunnett’s test (**e**). In **d**, statistical significance was determined by pairwise Fisher’s exact tests that compared each treatment to the control which was AX: Cleared. Exact *p* values are indicated in each panel of the figure.

The sugar meals adult females were fed contained no B vitamins while vertebrate blood also contains low amounts of B vitamins^32^. We thus assessed whether the smaller clutch sizes AX females laid were due to B vitamin deficiency. Adding all of the B vitamins in H + LA to the sugar solutions AX adults were provided *ad libitum* resulted in females laying first and second clutch sizes that did not differ from CN females (Fig. 4a, b). While loss of riboflavin, thiamin or pyridoxine inhibit *A. aegypti* larvae from pupating^26^, adding only these B vitamins to sugar solutions did not restore egg production to CN levels (Fig. 4a). Omitting folate from H + LA also earlier reduced the proportion of AX larvae that developed into adults^26^ while adding folate to an undefined diet increased the proportion of larvae that emerged as adults after clearance of an *E. coli* auxotroph^18^. However, provisioning only folate to AX females resulted in first clutch sizes that were smaller than those laid by CN females (Fig. 4a). Thus, AX females required multiple B vitamins to lay egg clutches that did not differ from those of CN females.

The AX and CN adults used in the preceding assays were reared as similarly as possible, but nonetheless differed in terms of microbial exposure. We therefore also assessed whether reintroducing bacteria to AX adults produced by clearance of *E. coli* also increased clutch sizes. As earlier noted, we had previously isolated several members of the CN community in our laboratory *A. aegypti* culture^16,22,24^. From these, we selected four species (*Serratia* sp. named strain UGAL515B_01, *Delftia* sp. UGAL515B_02, *Acinetobacter* sp. UGAL515B_03 and *Sphingobacterium* sp. UGAL515B_04) that earlier results showed to support development into adults when added to AX larval cultures^22,24^. We sequenced each using the PacBio platform, which yielded complete genome assemblies, predicted coding sequences (CDSs) and other metrics (Supplementary Table S1). Metabolic network reconstruction predicted *Serratia* sp. is a complete autotroph for all of the B vitamins in H + LA (Supplementary Fig. S3) which as earlier noted is also the case for *E. coli* K12^26^. *Sphingobacterium* sp., *Acinetobacter* sp, and *Delftia* sp. were predicted to have complete de novo synthesis pathways for riboflavin, thiamin, pantothenate, biotin, and folate but lacked genes in the canonical deoxyxylulose-5-phosphate (DXP) dependent and independent pathways for biosynthesis of pyridoxine (Supplementary Fig. S3). However, some bacteria also recycle and/or use incompletely understood alternative routes for pyridoxine biosynthesis^52,53^, which suggest these species could also provision this vitamin. We additionally did not identify homologs for *nadD* in *Acinetobacter* sp. or *Delftia* sp., which is required for converting nicotinate to NAD^+^ or NADP^+^ but *A. aegypti* encodes this gene, which suggested it uses nicotinate from bacteria or dietary sources (Supplementary Fig. S3). In contrast, *A. aegypti* lacks most other genes required for synthesis of the B vitamins in H + LA (Supplementary Fig. S3). Individually adding each of these bacteria to the sugar solutions AX adults were provisioned on the day of emergence resulted in each persisting in the gut for 9 days in females that were blood fed on day 4 post-emergence (Supplementary Fig. S4). Abundances were relatively constant for some species while others increased to 10^6^−10^8^ CFUs after blood feeding before declining by 9 days to densities that were similar to before blood feeding (Supplementary Fig. S4). Females provisioned with these bacteria also laid larger clutches than AX females with the possible exception of *Delftia* sp. as did CN females (Fig. 4c). Unexpectedly though, a higher proportion of females hosting *E. coli* K12, *Delftia* sp., and *Acinetobacter* sp. died after laying a first clutch when compared to AX females (Fig. 4d). This finding suggested these bacteria adversely affected adult females that blood fed although none showed adverse effects in earlier studies conducted in larvae^16,22,24^. Since the *Serratia* sp. did not reduce adult survival and was also predicted to be a complete B vitamin autotroph, we measured second clutch sizes laid by females hosting this bacterium which showed that clutch sizes were also similar to those laid by CN females but were larger than those laid by AX females (Fig. 4e). Thus, provisioning AX females with B vitamins or reintroducing B vitamin autotrophs both increased clutch sizes to levels laid by CN females.

### Axenic adults exhibit lower metabolic rates but longer lifespans that are also restored by B vitamin provisioning

**Fig. 5:**
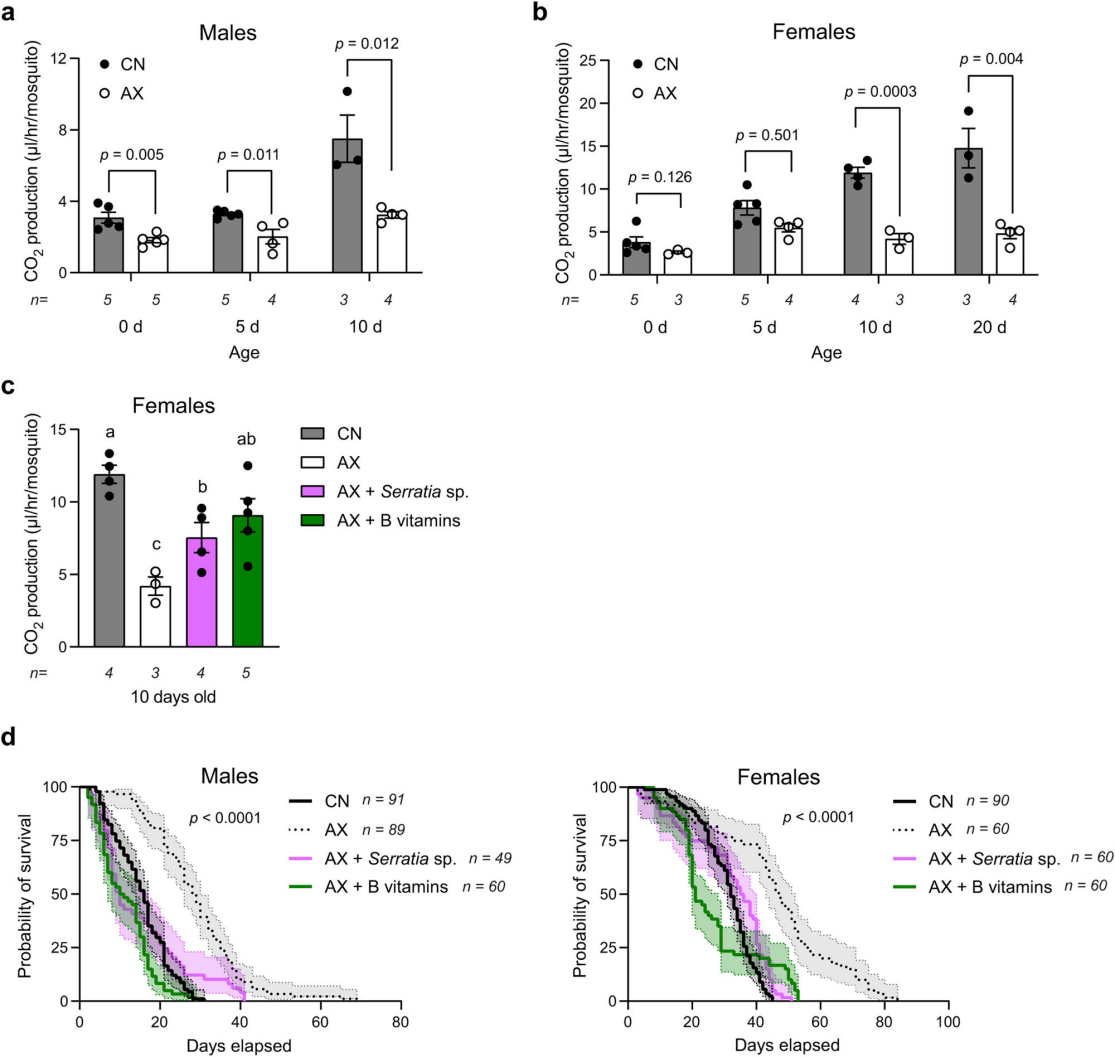
AX mosquitoes exhibit lower metabolic rates and longer lifespans than CN females. **a** CO_2_ production by 1, 5, or 10 day old CN males or AX males produced by clearance of *E. coli*. **b** CO_2_ production by 1, 5, or 10 day old CN females or AX females produced by clearance. **c** CO_2_ production by 10 day old: CN females, AX females produced by clearance, AX females produced by clearance and inoculated day 1 post-emergence with *Serratia* sp. (AX + *Serratia* sp.), or AX females produced by clearance and provisioned with all of the B vitamins in H + LA medium (AX + B vitamins). **d** Kaplan–Meier plots showing survival of adult males and females for the same treatments as in **c**. In **a**–**c**, data show mean ± SE with exact number of independent replicates analyzed for each treatment and time point below the x axis of each graph. Each replicate consisted of measuring CO_2_ production by 10 pooled males or 5 pooled females. Statistical significance was determined by pairwise Student’s *t* test using two-stage step-up false discovery rate corrections (**a**, **b**) or assessment of homogeneity of variances followed by one-way ANOVA and a Tukey–Kramer multiple comparison test (**c**). In **d**, data show survival proportions ± 95% confidence intervals with exact numbers of individuals analyzed per treatment indicated in the figure legend. Statistical significance was determined by log-rank (Mantel–Cox) tests which indicated survival curves significantly differed among treatments due to increased longevity of AX males and females. Exact *p* values are indicated in each panel of the figure.

In the field, *A. aegypti* spends long periods resting on walls or other surfaces and is thought to only fly short distances over its lifetime^54^. Activity assays in the laboratory similarly indicate adults spend ~75% of their time on average resting^55^. We also observed predominant resting and infrequent flight for both AX and CN adults. For most organisms including insects, O_2_ input directly correlates with CO_2_ output which reflects overall metabolic levels that can be compared if activity levels do not differ between treatment groups^56^. We thus established cages in which AX adults produced by clearance of *E. coli* or CN adults were provided *ad libitum* access to water and sucrose. We then measured CO_2_ production in resting adults of increasing age as a proxy for metabolic rate. AX males produced less CO_2_ than CN males at each sample time, whereas AX females produced less CO_2_ than CN females at 10 and 20 days old (Fig. 5a, b). In contrast, provisioning the B vitamins in H + LA to AX adults through sugar solutions resulted in 10 day old AX females producing CO_2_ levels that did not differ from CN females (Fig. 5c). Inoculating AX females produced by clearance with the *Serratia* sp. UGAL515B_01 that rescued egg production resulted in 10 day old females that produced more CO_2_ than AX females but less than CN females (Fig. 5c). Since metabolic rates were overall lower in AX than CN adults but increased with B vitamin provisioning, we also measured survival rates. Strikingly, AX males and females lived longer than CN males and females, whereas provisioning B vitamins or reintroducing *Serratia* sp. resulted in survival curves that did not differ from CN adults (Fig. 5d, e).

### Axenic females exhibit lower DENV vector competence

*A. aegypti* is the primary vector of dengue virus (DENV), which annually infects more than 100 million humans worldwide^57^. Previous studies report that certain commensal bacteria in larval cultures have carryover effects that reduce DENV vector competence in CN adults by unknown mechanisms^17,38^, while short-term pretreatment of CN adults with antibiotics increased vector competence as measured by higher DENV midgut titers 7 days post-infection (dpi)^40^. This latter outcome was further hypothesized to occur because bacteria in the gut prime the immune system to increase defense against arboviruses^58–61^, whereas treatment with a cocktail of antibacterial agents reduced microbiota abundance in the gut and associated defense^40^. However as earlier noted, studies also report that resistant bacteria and other microbes like fungi that are non-susceptible to antibacterial agents are commonly present in CN mosquitoes that are not cleared by antibiotic treatment^41,42^.

**Fig. 6:**
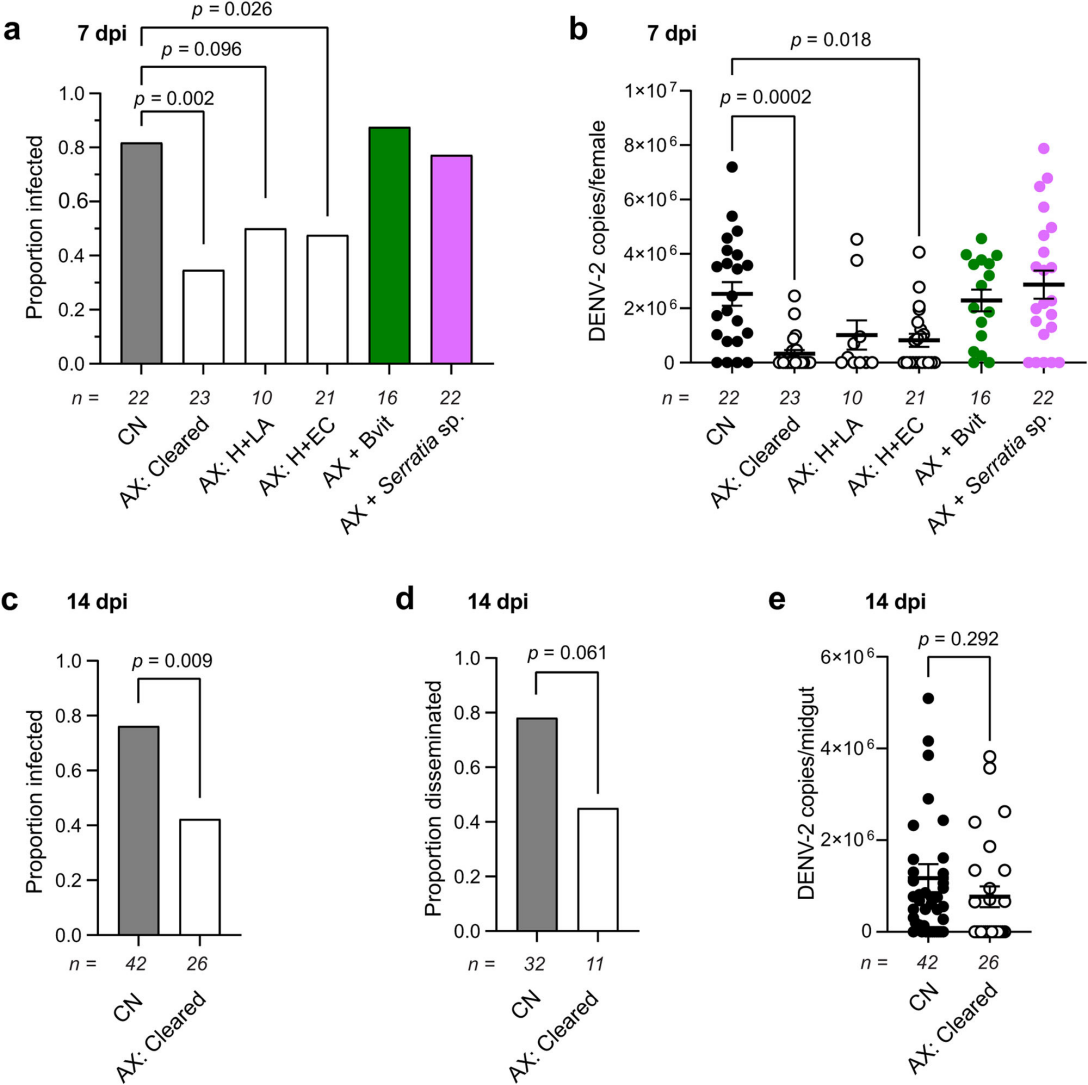
AX females exhibit lower DENV-2 vector competence than CN females. **a** Proportion of CN females, AX females produced by clearance of *E. coli* K12 (AX: Cleared), or AX females produced by axenic rearing (AX: H + LA or AX: H + EC) with detectable DENV-2 in the midgut at 7 days post-infection (7 dpi). Additional treatments were AX females produced by clearance that were provisioned with all of the B vitamins present in H + LA (AX + Bvit) or inoculated with *Serratia* sp. on day 1 of emergence (AX + *Serratia* sp). **b** DENV-2 genome copies per female at 7 dpi for the same treatments as in (**a**). **c** Proportion of CN females and AX females produced by clearance with detectable DENV-2 in the midgut at 14 dpi. **d** Proportion of CN females and AX females produced by clearance with a disseminated DENV-2 infection at 14 dpi. **e** DENV-2 genome copy number in the midguts of CN females and AX females produced by clearance at 14 dpi. In **a**, **c** and **d**, the exact number of females analyzed for each treatment is indicated below the x axis of each graph. Statistical significance was determined by pairwise Fisher’s exact tests that compared each treatment to the control which was CN. In **b** and **e**, data show mean ± SE with the exact number of females analyzed for each treatment indicated below the x axis of each graph. Statistical significance was determined after assessment of homogeneity of variances followed by a Kruskal–Wallis and post-hoc Dunn’s test (**b**) or a Welch’s *t*-test (**e**). Exact *p* values are indicated in each panel of the figure.

Given this background, we first asked if vector competence of AX females produced by clearance of *E. coli* or axenic rearing exhibited differences in DENV vector competence relative to CN adults. The potential for carryover effects also prompted us to produce AX adults by a third method that replaced lactalbumin in H + LA with autoclaved *E. coli* K12 (H + EC) that similarly supports development into adults if maintained in darkness but also results in larvae contacting dead bacteria during growth^26^. AX and CN adults were then fed infectious blood meals containing DENV type 2 (DENV-2) at a titer of 1 × 10^6^ TCID_50_ per ml, and following an extrinsic incubation period of 7 days, total viral load per tissue was estimated by RT-qPCR that measured genome copy number. Infection prevalence and genome copy number was higher in CN adults than AX adults produced by clearance or axenic rearing on H + Ec (Fig. 6a, b). In contrast, AX females produced by clearance that were provisioned with B vitamins in sugar solutions or by introducing *Serratia* sp. UGAL515B_01 exhibited infection prevalences and DENV genome copy numbers after a 7 day extrinsic incubation period that were similar to CN females (Fig. 6a, b). *Serratia marcescens* is an opportunistic pathogen of many insects^62^, but has also been shown to promote DENV infection of *A. aegypti* through a secreted protein named enhancin that digests mucins lining the midgut^63^. Earlier studies of another *Serratia* sp. also describe a protein with similar properties to enhancin^64^. However, phylogenetic analysis indicated *Serratia* sp. UGAL515B_01 is not closely related to *S. marcescens* (Supplementary Fig. S5), and does not encode an enhancin homolog. Extending our analysis of AX females produced by clearance to 14 dpi indicated infection prevalence remained lower than in CN females (Fig. 6c). Prevalence of a disseminated infection to the head and legs, which served as a proxy for transmission potential, also trended lower in AX than CN females (Fig. 6d), but we detected no difference in DENV-2 genome copy number between AX and CN females for those females with a disseminated infection (Fig. 6e). Overall, these results indicated DENV-2 vector competence was lower in AX than CN adults but increased in AX females that were provisioned with B vitamins or a B vitamin autotroph (*Serratia* sp. UGAL515B_01) lacking an enhancin homolog.

The preceding outcomes were also opposite results where CN adults pretreated with antibiotics exhibited increased DENV competence^40^. We thus repeated the same antibiotic regimen (AB 1x) with CN females from our own *A. aegypti* culture followed by feeding a high titer infectious blood meal (10^6^ TCID_50_/mL). Almost no mortality occurred in untreated CN females, whereas pretreatment with AB 1x resulted in 56% mortality by 5 dpi and 78% mortality by 14 dpi suggesting off target effects (Supplementary Fig. S6a), which had earlier also been observed when CN larvae were treated with antibiotics^41^. Reducing antibiotic concentrations 10-fold (AB 1/10x) resulted in no significant differences in mortality when compared to untreated females (Supplementary Fig. S6a). We also altered conditions by lowering DENV titer of the infectious meal to 10^4^ TCID_50_/mL and found that this modestly improved AB 1x survival (Supplementary Fig. S6b). We therefore used low titer infectious blood meals to investigate effects of antibiotic treatment on *A. aegypti* DENV competence. However, because of the continued high mortality of females treated with AB 1x, we only assessed the proportion of females that were DENV-infected at 5 dpi. A higher proportion of *A. aegypti* treated with AB 1/10x were infected with DENV-2 but no difference in infection prevalence was detected for the AB 1x treatment when compared to CN control females (Supplementary Fig. S6c). DENV-2 titer per female in the midgut was also modestly higher in the AB 1/10x treatment females but no difference in titer was detected between females treated with AB 1x and the CN control (Supplementary Fig. S6d).

## Discussion

Our interest in conducting this study stemmed from the literature which suggests facultatively hematophagous insects like mosquitoes avoid nutrient deficiencies by carryover of B vitamins and potentially other nutrients from larvae^32^. However, other results indicate animals including mosquitoes do not store B vitamins^26,33^, while prior studies of carryover effects in mosquitoes have used non-sterile adults with microbes that could provision B vitamins^17,34–38^. We therefore produced AX adults by clearing GN larvae of *E. coli* K12 which is a B vitamin autotroph that fully supports growth of larvae into adults^16,26^. We also produced AX adults by axenic rearing given evidence suggesting microbes present during the larval stage can affect adult fitness traits^38^. We then compared these treatments to CN adults produced from larvae with a microbiota from our *A. aegypti* culture because the latter provided a reference for ‘normal’ function and other studies of carryover effects in the literature that used non-sterile adults. We also reared this CN treatment as similarly as possible to minimize differences with the AX treatments.

Our results show that clutch sizes and adult longevity do not differ between the AX treatments we generated but do substantially differ from the CN treatment. The clutch sizes laid by CN females in this study are also very similar to the clutch sizes females in our laboratory culture lay^65,66^. We thus hypothesized the B vitamins *E. coli* K12 and H + LA provision larvae do not carry over to the adult stage which would be consistent with earlier results indicating *A. aegypti* does not store riboflavin^26^. Our finding that provisioning B vitamins to AX adults restores clutch sizes and longevities to CN levels also supports this hypothesis while also supporting other studies indicating vertebrate blood is B vitamin deficient^32^. We recognize though rearing conditions, while very similar, were not fully identical for our AX and CN treatments. We therefore also used AX adults produced by clearance to assess whether clutch sizes increase if *E. coli* K12 is reintroduced or conventional community members that are also B vitamin autotrophs are introduced. Results indicated clutch sizes increased when compared to the AX control but were very similar to CN adults or AX adults provisioned with B vitamins. Taken together, these findings strongly indicate carryover effects from the larval stage do not enable AX adults to function normally as compared to our CN treatment. That several B vitamin autotrophs increase clutch sizes comparably to B vitamin supplementation also strongly suggest adults require a gut microbiota under normal rearing conditions to provision B vitamins for normal function.

We are unclear why introducing *E. coli* K12, *Delftia* sp. and *Acinetobacter* sp. increased mortality after adult females blood fed and laid eggs in this study. Prior results implicate the gut microbiota in blood meal digestion while also showing that some bacteria increase in abundance in the gut after blood feeding^39^. Such increases could potentially underlie the increase in mortality we observed in the case of *Delftia* sp. and *Acinetobacter* sp. that both rose to more than 10^8^ CFUs per female after blood feeding which was approximately a log unit higher than the increase in total culturable bacteria detected in CN females (Supplementary Fig. S4). However, this pattern does not explain mortality associated with reintroducing *E. coli* K12 to AX adults as this species did not substantially increase after blood feeding. We also observed no increase in mortality in females with *Sphingobacterium* sp. that increased in abundance after blood feeding similarly to females hosting a CN community. Thus, other factors besides density underlie the increase in mortality we observed. However, these outcomes were why we restricted our measures of second clutch sizes, metabolic rates and DENV vector competence to introducing *Serratia* sp. UGAL515B_01 that is a complete autotroph for all of the B vitamins mosquitoes appear to require for development^26^.

The smaller clutches AX females laid in this study are associated with defects in vitellogenesis but our finding that AX females produce similar numbers of eggs as CN females when provisioned with B vitamins or bacteria that are B vitamin autotrophs suggest defects in vitellogenesis are due to B vitamin deficiencies. Earlier studies in larvae indicate B vitamin deficiencies also cause a number of physiological defects that are most severe in the absence of riboflavin which prevents larvae from growing or molting^26^. Larvae likewise arrest if reared with an *E. coli* K12 auxotroph that cannot produce riboflavin, whereas adding riboflavin to the diet or replacing this auxotroph with wild-type *E. coli* restores normal growth^26^. However, the loss of other B vitamins including thiamin, pyridoxine and folic acid also cause growth defects in *A. aegypti* larvae^18,26^. In this study, provisioning all of the B vitamins in H + LA to AX adults increased egg clutch sizes to the numbers laid by CN adults, but provisioning only riboflavin pyridoxine and thiamin or only folic acid did not suggesting insufficient carryover from larvae combined with additional deficiencies in sugar and blood results in AX adults lacking other B vitamins that are required for normal egg production and longevity.

We have not conducted a metagenomic analysis of the community of bacteria present in our laboratory *A. aegypti* culture, but our isolation, sequencing and annotation of four community members that earlier studies showed to support development of AX larvae into adults^24^ indicates each is a complete or near complete B vitamin autotroph, which also strongly suggests the CN community as a whole can provision B vitamins to larvae and adults in our culture. Variable communities of bacteria have been identified from *A. aegypti* and other mosquitoes in the field and laboratory^11,67^. However, we think it likely most communities of microbes associated with mosquitoes contain bacteria that are partial or complete B vitamin autotrophs given the comparative literature which indicates many environmental bacteria in families and genera that are common community members in mosquitoes synthesize riboflavin and other B vitamins^68,69^. Our previous results also identify several bacteria from field and laboratory cultures that enable AX larvae to develop into adults which suggests these unsequenced species are also B vitamin autotrophs in light of evidence showing that wild-type *E. coli* K12 rescues growth of AX larvae but mutants defective for synthesis of, for example, riboflavin cannot^16,17,22,26,41^. However, a few studies do report that supplementing CN cultures with B vitamins enhances mosquito fitness as measured by development time, adult longevity and egg production, which suggests microbial communities may not always provision sufficient amounts of one or more B vitamins^70,71^. As earlier noted, mosquitoes are unknown to host any heritable microbes that function as nutritional symbionts. One strain of *Wolbachia* has been identified that likely provisions riboflavin and biotin to obligately hematophagous bedbugs^72^. While some mosquitoes host natural *Wolbachia* infections, no role for *Wolbachia* in nutrient provisioning has been identified^73^. We thus hypothesize the prevalence of partial or complete B vitamin autotrophs in the environments where mosquitoes live reduces selection pressure to evolve associations with heritable symbionts to provision B vitamins.

In a previous study, AX *A. aegypti* produced by clearing a strain of *E. coli* resulted in no differences in egg production after the first gonadotrophic cycle or longevity when compared to GN progeny that were not cleared^18^. A higher proportion of these AX and GN adults also oviposited than CN adults while the CN adults that oviposited laid smaller clutches than the AX and GN females^18^. However, we also note the proportion of CN females that oviposited after blood feeding (~20%) and the clutch sizes laid (30–40 eggs) by CN, GN, and AX adults in this study are much lower than the UGAL strain we used and *A. aegypti* cultures used in other studies where more than 90% of females oviposit and lay clutch sizes of 80–120 eggs^65,66,74^. Longevity of the AX and GN adults in this earlier study^18^ were also more similar to the AX than the CN or rescued AX adults in this study. However, the GN adults in this previous study were noted to host very small numbers of *E. coli* owing to low transstadial transmission while no rescue experiments were conducted^18^. We too have observed that only low densities of *E. coli* K12 are transstadially transmitted to GN adults using our UGAL strain of *A. aegypti*, which could potentially contribute to bacterial abundance being too low to increase B vitamins and clutch sizes. *A. aegypti* adults developing from CN larvae have also been reported to have longer lifespans than adults from GN larvae that were reared with a single a commensal microbe^38^. Interpreting this finding in the context of this study though is difficult because adults were non-sterile and thus cannot be compared to the AX adults we assessed.

Resource constraints often promote trade-offs between reproduction and longevity in animals^75,76^, which is broadly consistent with AX adults producing fewer eggs but living longer than CN adults. However, results from this study further indicate AX adults have lower metabolic rates than CN adults. This latter point is of interest because several B vitamins produce cofactors with essential functions in metabolism including riboflavin that is required for electron transport and ATP production by mitochondria^26^. Defects in mitochondrial function and lower metabolic rates have also been shown to increase longevity in model organisms like *Caenorhabditis elegans* through alterations in signaling processes^77,78^. Thus, a potentially important future direction for study is understanding how deficiencies in B vitamins like riboflavin affect mosquito metabolism and potential consequences for a range of physiological processes.

Similar to egg production and longevity, our results indicate DENV-2 vector competence is lower in AX than CN females but is also rescued by B vitamin supplementation or introduction of a B vitamin autotroph to the midgut. These outcomes suggest the gut microbiota in the adults used in this study increase DENV-2 vector competence by provisioning B vitamins deficient in sugar and blood meals which is consistent with studies indicating arboviruses like DENV substantially depend on host cell metabolism for replication^79,80^. The lower DENV-2 titers detected in AX females that developed from larvae with *E. coli* K12 versus without (see Fig. 6b) could suggest a carryover effect from the larval stage that further reduces DENV titers in AX adults at 7 dpi by unknown means. Our results showing that DENV vector competence is similarly increased by B vitamin provisioning or addition of a complete B vitamin autotroph like *Serratia* sp. UGAL515B_01 further favor the suggestion both treatments increase host metabolism which promotes viral replication. In contrast, our results do not indicate *Serratia* sp. UGAL515B_01 encodes an enhancin or is closely related to *Serratia marcescens* that increases DENV vector competence via the activity of enhancin^63^. In contrast, other commensal or heritable microbes like certain strains of *Wolbachia* strongly reduce DENV vector competence by still incompletely understood mechanisms^59–61^.

## Methods

### Ethics statement

No vertebrate animals were used in the study. For maintenance of the *A. aegypti* mosquito culture, adult females were blood-fed with or without DENV using membrane feeders and commercially purchased rabbit blood (Hemostat Laboratories) with protocols approved by the University of Georgia institutional Biosafety Committee protocol 2022-0047.

### Laboratory *A. aegypti* culture

The University of Georgia strain of *A. aegypti* (UGAL) was maintained in an insectary under standard conditions (27 °C, 70% relative humidity, and a 12 h light: 12 h dark photoperiod) as earlier described^65^. Larvae were reared in pans containing 1 L water and fed rat chow mix (RCM) diet consisting of ground rat chow pellets (LabDiet 5001), lactalbumin (Sigma), and torula yeast extract (Bio-Serve) mixed in a ratio of 1:1:1 by volume. Adults were held in cages with *ad libitum* access to cotton-wick feeders containing 10% sucrose in water. Adult females (4–6 days old) for the general culture were fed defibrinated rabbit blood (Hemostat Laboratories) in water-jacketed glass (Rutledge) feeders warmed to 37 °C with Parafilm® as the membrane to produce eggs that were hatched for colony maintenance 10–14 days after oviposition.

### Bacteria

Glycerol stocks of *Escherichia coli* str. K12 substrain MG1655 were earlier prepared^16^. Glycerol stocks were also prepared for *Serratia* sp. strain UGAL515_B1, *Sphingobacterium* sp. strain UGAL515_B2, *Acinetobacter* sp. strain UGAL515_B3, and *Delftia* sp. strain UGAL515_B4 that were identified from UGAL *A. aegypti*^24^. Each was cryopreserved at −80 °C and used in this study by streaking onto Luria-Bertani (LB) medium-agar plates with no antibiotics. Liquid cultures used to inoculate AX larvae (see below) were grown overnight from a single colony in antibiotic-free Luria-Bertani (LB) broth at 37° (*E. coli*) or 30 °C (all other bacteria used in assays) in shaking incubators. Cultures with an optical density of 1 at 600 nm contained ~1×10^9^ colony forming units per ml^24^. Water was collected from a rearing pan in the general culture containing fourth instars. After removal of debris by gentle centrifugation (200 × *g* for 1 min), the microbial community in this water was collected by centrifugation at 6000 × *g* for 15 min by discarding the supernatant and cryopreserving the remaining pellet at −80 °C after resuspension in sterile glycerol and water (1:1). This community of microbes was then used to produce the adults with a conventional (CN) microbial community used in bioassays.

### Whole genome sequencing of bacteria and reconstruction of B vitamin biosynthesis pathways

DNA was isolated by phenol/chloroform extraction from bacterial cells grown on LB agar at room temperature (Pacific Biosciences, https://www.pacb.com/wp-content/uploads/2015/09/SharedProtocol). After quantification by Nanodrop (Eppendorf) and quality checking by gel electrophoresis, DNA was sequenced using the PacBio Sequel system at the UGA Georgia Genomics and Bioinformatics Core. De novo assembly of the raw PacBio data was performed independently using Flye V2.8 for *Serratia* sp. UGAL515_B1 and *Sphingobacterium* sp. UGAL515_B2 or Canu v 2.1.1 for *Acinetobacter* sp. UGAL515_B3 and *Delftia* sp. UGAL515_B4 followed by quality assessment using QUAST v5.02 and annotation using the PGAP pipeline^81–84^. RNAmmer and tRNAscan-SE was used for RNA annotation^85,86^. Kyoto Encyclopedia of Genes and Genomes (KEGG) pathway analysis was performed by annotating the genes in each bacterium we sequenced, *E. coli* K12, and *A. aegypti* with KO identifiers using the KEGG Automatic Annotation Server^87^. KEGG Mapper was then used to match genes with the pathway in the KEGG database to infer biosynthetic capabilities^88^. The basic local alignment search tool was used to assess whether a homolog for *S. marcescens* enhancin^63^ was present in the genome of *Serratia* sp. UGAL515_B1.

### Axenic (AX) and conventional (CN) cultures

AX and CN adults were reared as larvae under identical conditions in 6 well culture plates (Corning) (Supplementary Fig. S1). Ten-14 day old eggs from the general culture were surface-sterilized as earlier described^16^ with the exception that 0.1% Roccal (Pfizer) was replaced with 0.1% D-256 (Vedco). Surface sterilized eggs were transferred to a 10 cm^2^ culture flask (Corning) containing sterile water where AX first instars hatched. To produce AX adults from larvae that were cleared of *E. coli*, 5 AX first instars were placed 6-well culture plates (Corning) in which each well contained 5 ml of sterile water. Sixty μl of *E. coli* K12 (~10^8^ CFUs) from an overnight culture was then pelleted and resuspended in sterile water before adding to the well. Resulting GN larvae were fed RCM diet that had been sterilized by gamma irradiation to 10 kGy under standard conditions (Supplementary Fig. S1)^16^. Although unnecessary for growth, GN larvae were reared in darkness to be consistent with rearing of larvae in AX cultures (see below). Ampicillin (Sigma) was added at 100 ug/ml to each culture well 48 h after GN larvae had molted to the fourth instar, which was followed by pupation the next day. Pupae were collected, surface sterilized using 2% bleach, and transferred to cups containing sterile water that were placed in sterile nested containers where AX adults emerged (Supplementary Fig. S1). To produce AX adults without exposure to living bacteria, five AX first instars from surface sterilized eggs were added per culture well containing 5 ml of sterile water and fed H + LA or H + EC diet in darkness as earlier described^26^. We fed AX larvae these diets because prior results indicated RCM diet is riboflavin deficient^26^. Resulting pupae were transferred to cups containing sterile water that were placed in sterile nested containers similarly to AX adults that were produced by clearance of *E. coli* (Supplementary Fig. S1). CN adults were produced by placing five AX first instars from surface sterilized eggs per culture well containing 5 ml of sterile water that was inoculated using a sterile loop with the mixed community of microbes in the glycerol stock prepared from the general culture (see above). CN larvae were then fed gamma irradiated RCM diet and maintained in darkness to be consistent with GN and AX larvae until pupation. Resulting pupae were transferred to cups containing sterile water that were placed in nested containers as used for AX adults (Supplementary Fig. S1). All adult mosquitoes used in bioassays were provided *ad libitum* access to sterile water or 10% sucrose solutions that were replaced daily. Females were blood fed using defibrinated, sterile rabbit blood in small-scale membrane feeders^60^ that were surface sterilized using 70% ethanol (3 min), povidone-iodine (Betadine) (3 min), and 70% ethanol (3 min) before air drying. Females were allowed to feed for 30 min or 1 h depending on assay (see below).

### Sterility assessment

Culture- and polymerase chain reaction (PCR)-based methods were used to screen for sterility in all cohorts of AX larvae hatched from surface sterilizing eggs or AX adults used in bioassays. We also screened all cohorts of GN or CN progeny. Larvae or adult samples were homogenized (whole-body without external sterilization) in sterile phosphate-buffered saline (PBS) and spread onto antibiotic-free LB-agar plates that were incubated at 30 °C for three days to confirm either no microbial growth (AX adults), colonies with morphology corresponding to *E. coli* K12 (GN progeny), or colonies with different morphologies (CN progeny). PCR assays were conducted to assess status by isolating total DNA from mosquitoes followed by reactions using universal primers 27 F (5’-GAGAGTTTGATCCTGGCTCAG-3’) and 1492 Rv (5’-GGTTACCTTGTTACGACTT-3’) that amplify a portion of the bacterial 16 S rRNA gene or near-universal primers ITS1-F (5’-CTTGGTCATTTAGAGGAAGTAA-3’) and LR3 (5’-CCGTGTTTCAAGACGGG-3’) that amplify a fungal internal transcribed spacer (ITS) region^89^. PCR products were visualized on 1% agarose gels stained with ethidium bromide, run at 90 V for 45 min (LabNet Gel Electrophoresis System). Detection of amplicons in cohorts from larvae inoculated with *E. coli* K12 or microbes from CN glycerol stocks together with appropriate colony morphologies on culture plates were classified as GN or CN mosquitoes while extracts that generated no visible 16 S or ITS amplicons or colonies were classified as AX mosquitoes (Supplementary Fig. S2). Contamination was infrequent but any cohort that deviated from these outcomes was discarded. For adults that were provided *E. coli* K12 or *Serratia* sp. in sugar solutions, 4 females per treatment at 3 days post-inoculation, 24 h post-blood meal (4 d post-inoculation), and 4 days post-bloodmeal (8 d post-inoculation) were sampled for whole-body CFU counts by plating *E. coli-*fed mosquito homogenates on LB-agar and *Serratia* sp.-fed mosquito homogenates on tryptic soy agar (TSA).

### Development metrics

Development time to pupation (days) was assessed for GN progeny with *E. coli* K12, AX progeny produced by clearance of *E. coli* K12 using ampicillin, or CN progeny with at least three different rearing cohorts consisting of at least 10 individuals per replicate being measured. Sex ratio was determined by tallying number of males and dividing by the total number of adults per replicate. Wing length of adults was used as a proxy for body size and was assessed by removing and slide-mounting the one wing per adult and measuring the length in mm from the alular notch to the wingtip apex^22,24^. Adult survival was tracked daily beginning from 24 h post-eclosion until all individuals were dead for 3 independent replicate cages that each contained 20–30 males or females; both sexes were provided sterile 10% sucrose *ad libitum* and females were additionally blood-fed on day four. For the duration of survival assays all adults were held in a climate-controlled incubator under standard conditions.

### Blood feeding and clutch sizes

The proportion of AX females produced by clearance and CN females that blood fed was assessed by providing membrane feeders to females and counting the number of engorged individuals after 1 h divided by the total number females present. The amount of ingested blood per female was estimated by cold-anesthetizing and weighing 5 cohorts of 10 CN females or 8 cohorts of 10 AX females using an analytical balance (Ohaus DV215CD) to generate an average NBF mass for each replicate. Females were given 8 h to recover, blood fed, and replete females were isolated and weighed, again pooled by replicate, within 15 min to generate median, minimum and maximum blood meal weights (mg) per female.

Clutch sizes laid by adult females were assessed by provisioning filter-sterilized water and sugar solutions *ad libitum*. All B vitamins at the same working concentration as in H medium^26^ (thiamin 268 ng/ml, riboflavin 990 ng/ml, nicotinic acid 1 ug/ml, pyridoxine 1.5 ug/ml, calcium pantothenate 332 ng/L, biotin 28 ng/L, and folic acid 300 ng/ml)^26^ were provisioned to AX females by adding them daily to sugar solutions. Bacteria from cultures grown in Luria-Bertani broth were pelleted by centrifugation, rinsed 2X in sterile water and added to sugar solutions at a density of 10^6^ CFUs/ml that were provisioned to newly eclosed AX females produced by clearance for three days. For each treatment, females were blood fed on day 4 post-emergence for a first gonadotrophic cycle and day 8 post-emergence for a second gonadotrophic cycle using defibrinated, sterile rabbit blood in small-scale membrane feeders as described above. Females were allowed to feed for 30 min. First and second clutch sizes were determined by selecting females from four independent rearing cohorts and placing them in sterile, polypropylene cups with screen lids (2.86 cm×2.86 cm, US Plastics) that contained sterile wetted paper as an oviposition site. At 3 d post-inoculation, 24 h post-blood meal, and 4 d post-bloodmeal ( = 9 days old), four females per treatment were sampled for whole-body CFU counts using culture-based methods^90^. *E. coli*-fed mosquito homogenates were plated on LB-agar, *Delftia* sp.-fed mosquito homogenates on 869 medium while the other treatments including the sterile AX control were cultured on tryptic soy agar.

### Vitellogenesis assays

Activation of the vitellogenic phase in the first and second gonadotrophic cycle was assessed by measuring ecdysteroid production by the ovaries, yolk deposition into oocytes, and vitellogenin production by the fat body. Ecdysteroids were quantified using a previously established ex vivo enzyme immunoassay^91^. Samples were generated by incubating paired ovaries from two females were collected by dissection at 20 h PBM and cultured in 60 µl of Beyenbach’s saline for 6 h at 27 °C and ~90% RH. The ecdysteroids secreted into the saline solution were then determined by EIA using primary antibody EAB27 and 20E standards (Sigma) with 7–15 biological replicates obtained for each treatment and time point^91^. Yolk deposition into oocytes was measured in μm along the anterior-posterior axis as illustrated in Fig. 2b, averaged for three representative oocytes per female with at least 5 females measured from three independent rearing cohorts. Vitellogenin was visualized by collecting ovaries from two females and remaining abdomens containing the fat body in Pro-Prep™ (iNtRON Biotechnology) plus 10x Halt™ protease and phosphatase inhibitor cocktail (ThermoFisher). Following homogenization, sonication, and centrifugation to remove debris, protein extracts were mixed with Laemmli sample buffer (Bio-Rad) containing 5% ß-mercaptoethanol and boiled for 10 min. Samples contained pooled tissues from two individuals, while a 1/5 tissue equivalent for fat body samples and a 1/10 tissue equivalent for ovary samples were loaded per lane for each treatment. Samples were electrophoresed at 100 V for 1 h using 4–20% Tris-glycine polyacrylamide gels (Mini-PROTEAN TGX stain-free, Bio-Rad), then transferred to polyvinyl difluoride (PVDF; ThermoFisher) using 0.35 A for 1 h. Blots were blocked in 5% nonfat dry milk in PBS with 0.1% Tween 20 for 1 h, then probed with rabbit anti-Vg (1:80,000) in milk block overnight. Following three washes in PBS with 0.1% Tween 20, blots were probed with a peroxidase-conjugated goat anti-rabbit secondary antibody (1:5,000; Jackson) in milk block for 2 h followed by application of Clarity™ ECL chemiluminescent substrate (Bio-Rad) and imaging (Syngene G:Box). Immunoblots were repeated in triplicate using independently collected samples.

Relative transcript abundance of late trypsin-like genes was analyzed by real-time quantitative reverse transcriptase PCR (rqRT-PCR) using total RNA from 24 h PBM midguts (two midguts per replicate, 4 biological replicates per treatment) extracted in Trizol reagent (Ambion). cDNA was generated using iScript (Bio-Rad) while gene specific primers were designed and purchased (IDT) for: *Aedes aegypti early trypsin* (*AaET*, X64362.1; forward 5’-ACCGTGGCAGATGGAGCTATG-3’; reverse 5’-GGCATAACCAGCGCAGATCAT-3’; *Aedes aegypti late trypsin* (*AaLT*, M77814.1; forward 5’-GGAAGTGATACCTTTACCGACCG-3’; reverse 5’-GATCACCAACGGGCTGTAGGC-3’), and *Aedes aegypti serine protease VI (5G1)* (*AaSPVI*, GQ398048; forward 5’-AGGAATGCCACAAGGCTTACTTGA-3’; reverse 5’-CCATAACCCCAGGATACCACT-3’) (35). *Aedes aegypti actin* (*AaACT*, KY000701) was used as reference gene using primers forward 5’-CGTTCGTGACATCAAGGAAA-3’ and reverse 5’-GAACGATGGCTGGAAGAGAG-3’^31,58^. Reactions contained 3 µl cDNA, 2 µl forward/reverse primers 5 µM, and 5 µl iQ SYBR Green Supermix (Bio-Rad 170-8882) and were run using a Rotor-Gene Q real-time PCR cycler (Qiagen) under the following conditions: denaturation at 95 °C for 10 sec and annealing at 60 °C for 45 sec, for a total of 30 cycles. Relative transcript abundance of each target gene relative to *AaACT* and fold change compared to non-fed controls was calculated using the ΔΔCT method^51^.

### Triacylglycerol and lipid droplet quantification

TAG stores in the fat body of previtellogenic and vitellogenic females was measured by collecting abdomens with the gut and ovaries removed (pelts) from day 4 females immediately post blood feeding (=0 h PBM) and females at 24 h intervals up to 72 h PBM^51^. Each biological sample consisted of two pelts that were homogenized in 100 µl of PBS containing 0.5% Tween-20 and then incubated at 70 °C for 5 min. Pelt samples were centrifuged at 3000 × *g* for 1 min and 15,900 × *g* for 3 min followed by transfer of 10 µl from each sample to individual wells of 96-well plates (Corning). After adding 100 µl of TAG reagent (Thermo-Fisher) and gentle mixing, samples were incubated for 10 min at room temperature and absorbance was measured with a Synergy plate reader (BioTek) at 530 nm. A range of TAG standards (MedTest Dx) was included in the wells of each plate and used to calculate experimental values from a regression line. Lipid droplet accumulation in cells was assessed by fixing tissues in 4% paraformaldehyde in PBS for 20 min, two 5-minute rinses in PBS, permeabilization in PBS-Triton X-100 2% for 20 min, and staining with Nile Red 100 µg/mL (Molecular Probes) for 20 min. After washing in PBS for 10 min, samples were counterstained with Hoescht 33342 1 µg/mL for 10 min, slide-mounted in 1:1 glycerol-PBS and imaged using a Zeiss LSM 710 confocal microscope. The number of lipid droplets per cell was quantified for 5 representative cells per tissue sample to generate an average. A minimum of 7 independently collected tissue samples were analyzed per treatment and time point.

### Metabolic rates

CO_2_ production by resting adults was measured by respirometry using respirometers as designed by Yatsenko et al.^92^. Each biological replicate consisted of placing 5 females or 10 males that were 1, 5, 10 or 20 days old per respirometry chamber that was placed in a climate-controlled room (25 °C) for a minimum of 30 min before starting an assay. CO_2_ output was recorded as µl/mosquito/h. A minimum of 3 independently collected biological replicates were conducted for each treatment.

### Vector competence

Vector competence assays were conducted used DENV type 2 (DENV-2) originally obtained from the World Reference Center for Emerging Viruses and Arboviruses at the University of Texas Medical Branch (PRS 225 488, Thailand 1974). Viral stocks were propagated in Vero (African green monkey kidney epithelial) cells obtained from the American Type Culture Collection using an MOI of 0.1 or 0.01 and virus-containing supernatant collected at 4–6 dpi and frozen at −80 °C prior to use in infectious feeds. DENV-2 stocks in Vero cell conditioned medium (Dulbecco’s Modification of Eagle’s Medium +10% fetal bovine serum) were diluted in defibrinated rabbit blood at 1:10 to produce high-titer feeds or at 1:200 to produce low-titer feeds. The viral stock aliquot used and samples of infectious blood taken before and after each infectious feed were all titered by tissue culture infectious dose (TCID_50_) assay in Vero cells. Infectious titer was calculated using the Spearman-Karber method^93^ and was typically 10^6^ TCID_50_/mL for high titer feeds and 10^4^ TCID_50_/mL for low titer feeds, with infectious blood viral titer decreasing by one log unit over the course of the feed (45 min–1 h).

Four-day old adult females were offered infectious bloodmeals using water-jacketed glass (Rutledge) feeders warmed to 37 °C with Parafilm® as the membrane and 1 mM ATP added as a phagostimulant. Females that fed to repletion were isolated after the blood meal and held for either 7 d to determine infection prevalence and viral genome copy number in the midgut or 14 d to assess infection prevalence, viral genome copy number in the midgut and the proportion of females with a disseminated infection to the head and legs, which served as a proxy for transmission potential. Females were provided ad libitum access to water and 10% sucrose for the duration of the incubation period and maintained under standard conditions. Virus presence/absence and titer were measured by qRT-PCR as the large number of samples precluded titration by TCID_50_. Several pilot experiments revealed no viral dissemination at 7 dpi for any treatment; therefore, whole-body females were used to assess midgut infection at 7 dpi. Viral dissemination at 14 dpi was assessed in the pooled legs and head from each female, with the remaining thorax and abdomen (containing the gut) used to estimate titer. Sufficient numbers of females in each treatment were offered infectious blood meals to obtain a minimum of 30 replete females per treatment at 7 dpi or 14 dpi. Total RNA was extracted and cDNA was synthesized as above, while qRT-PCR reactions were run with DENV-specific NS5 region primers (forward 5’-ACAAGTCGAACAACCTGGTCCAT-3’; reverse 5’- GCCGCACCATTGGTCTTCTC −3’) (80) using a two-step PCR program as described above. The NS5 fragment that was PCR amplified from a DENV-2 stock cDNA template was ligated into a PCR®2.1 TOPO® TA (Invitrogen), transformed into One Shot™ TOP10 chemically competent cells (Invitrogen) and plasmid DNA was extracted using a GeneJET Plasmid Miniprep Kit (Thermo Fisher). Sanger sequencing (Eurofins Genomics) verified identity. Serial dilutions of the plasmid 10^9^−10^2^ copies/µl were then used to generate a standard curve by qRT-PCR which was used to estimate titer in each treatment sample which was determined from the average of four technical replicates.

### Statistics and reproducibility

Individual mosquitoes served as the unit of replication for most assays. Exceptions were digestive enzyme expression, ecdysteroids produced by ovaries, and TAG stores where each replicate consisted of dissected tissues from two or more mosquitoes or in the case of CO_2_ production five pooled adult female mosquitoes or ten pooled adult male mosquitoes. Proportional data sets were analyzed by contingency table analysis using Fisher’s exact test or Chi-square tests. Quantitative data sets involving two treatments including development time, wing length, and digestive enzyme expression were analyzed using an unpaired two-tailed Student’s t-tests or the nonparametric alternative Welch’s t-test if treatment variances were first determined unequal by F tests of variances. Grouped data comparing two treatments over a time course including ovary ecdysone production, yolk deposition, and CO_2_ production were analyzed using pairwise t-tests for which the two-stage step-up correction method for false discovery rate was applied. TAG levels and lipid droplets per cell data sets were both analyzed using a linear repeated measures mixed effects model. For TAG levels, replicate was assigned as a source of random variation, while for counting lipid droplets, individual cells and the tissue sample (from which they came were both assigned as sources of random variation followed by a nested sampling design. Clutch sizes and DENV-2 genome copy numbers were compared among treatments by analysis of variance (ANOVA). Data sets were first subjected to Bartlett’s test for homogeneity of variances and Shapiro-Wilk test for normality of residuals to determine if the data fit the assumptions of ANOVA. If data generated *p* values of greater than 0.05 for both tests, a one-way ANOVA was used to compare treatment means followed by either Tukey’s Honestly Significant Differences multiple comparisons test or by Dunnett’s post-hoc analysis where either CN or AX adults were designated as the positive or negative control respectively. If data were found to violate the assumptions of ANOVA due to either unequal variances among treatments or non-normal distribution of residuals, non-parametric Kruskal–Wallis tests were used followed by a Dunn’s post hoc multiple comparison test. Adult survival curves (Kaplin–Meier plots) were analyzed using logrank (Mantel–Cox) tests. Reinoculation of bacteria into AX adults and subsequent persistence in the gut over 9 days was assessed using a mixed effects repeated measures model. Analyses were conducted in R v.4.2.2 using the nlme package for linear mixed effects models or GraphPad Prism v.9.0.1. Figures were generated in GraphPad Prism followed by export to Adobe Illustrator (v.23.0.1) for sizing and additional labeling.

### Reporting summary

Further information on research design is available in the Nature Portfolio Reporting Summary linked to this article.

### Supplementary information

Supplementary Information

Reporting Summary

## Data Availability

The genomes for *Serratia* sp. (strain UGAL515B_01), *Spingobacterium* sp. (strain UGAL515B_02), *Acinetobacter* sp. (strain UGAL515B_03), and *Delftia* sp. (strain UGAL515B_04) are deposited in the National Center for Biotechnology Information (NCBI) GenBank in the Sequencing Read Archive (SRA) under BioProject ID PRJNA889817. All other data supporting the findings of this study are available within the paper and its Supplementary Information. The uncropped and unedited immunoblots used for Fig. 2e are provided in Supplementary Fig. S7.
