# Supplementary material for: The mosquito Aedes aegypti requires a gut microbiota for normal fecundity, longevity and vector competence
Source: Commun Biol. 2023 Nov 13;6:1154. doi: 10.1038/s42003-023-05545-z (PMC10643675; doi:10.1038/s42003-023-05545-z)
Supplement: Supplementary file 1 — Supplementary Information [file 42003_2023_5545_MOESM1_ESM.pdf]

Supplementary Information

The mosquito *Aedes aegypti* requires a gut microbiota for normal fecundity, longevity and vector competence

Ruby E. Harrison[1,2], Xiushuai Yang[1], Jai Hoon Eum[1], Xiaoyi Dou[1], Vincent G. Martinson[3], Luca Valzania[4], Yin Wang[1], Bret M. Boyd[5], Mark R. Brown[1], Michael R. Strand[1*]

Author Affiliations

[1]Department of Entomology and Center for Tropical and Emerging Global Diseases, University of Georgia, Athens, GA, 30602 USA

[2]Current address: Department of Cellular Biology and Center for Tropical and Emerging Global Diseases, University of Georgia, Athens, GA 30602 USA

[3]Department of Biology, University of New Mexico, Albuquerque, NM 87131 USA

[4]Institut Curie, 20 Rue d'Ulm, 75238 Paris Cedex 05, France

[5]Center for Biological Data Science, Virginia Commonwealth University, Richmond, VA, 23284 USA

[*]Corresponding author: Michael R. Strand, Department of Entomology, University of Georgia, 120 Cedar Street, 420 Biological Sciences, Athens, GA, 30602 USA, mrstrand@uga.edu

Supplementary Table S1 Assembly statistics and genome features of sequenced bacteria.

Species	*Serratia* sp.	*Sphingobacterium* sp.	*Acinetobacter* sp.	*Delftia* sp.
Accession	CP109900	CP109907	CP109902	CP109906
Strain	UGAL515B_01	UGAL515B_02	UGAL515B_03	UGAL515B_04
Reads	924,825	1,960,788	402,407	580,362
Chromosome Size (bp)	4215431	6363396	3465529	6685457
Mean coverage	684X	952X	467X	419X
N50	8635	8209	8024	9320
GC %	48.7	39.9	43.2	66.6
Circularized	Yes	Yes	Yes	Yes
Plasmids (bp)	46300	No	13701, 13507	No
Coding density	83.3	87.8	87.7	90.1
CDS	3642	5217	3198	6043
Hypothetical CDS	330	1118	421	835
Average CDS size (bp)	964	1071	951	997
ncRNA	5	2	3	2
rRNA	22	21	21	15
tmRNA	1	1	1	1
tRNA	82	85	74	81

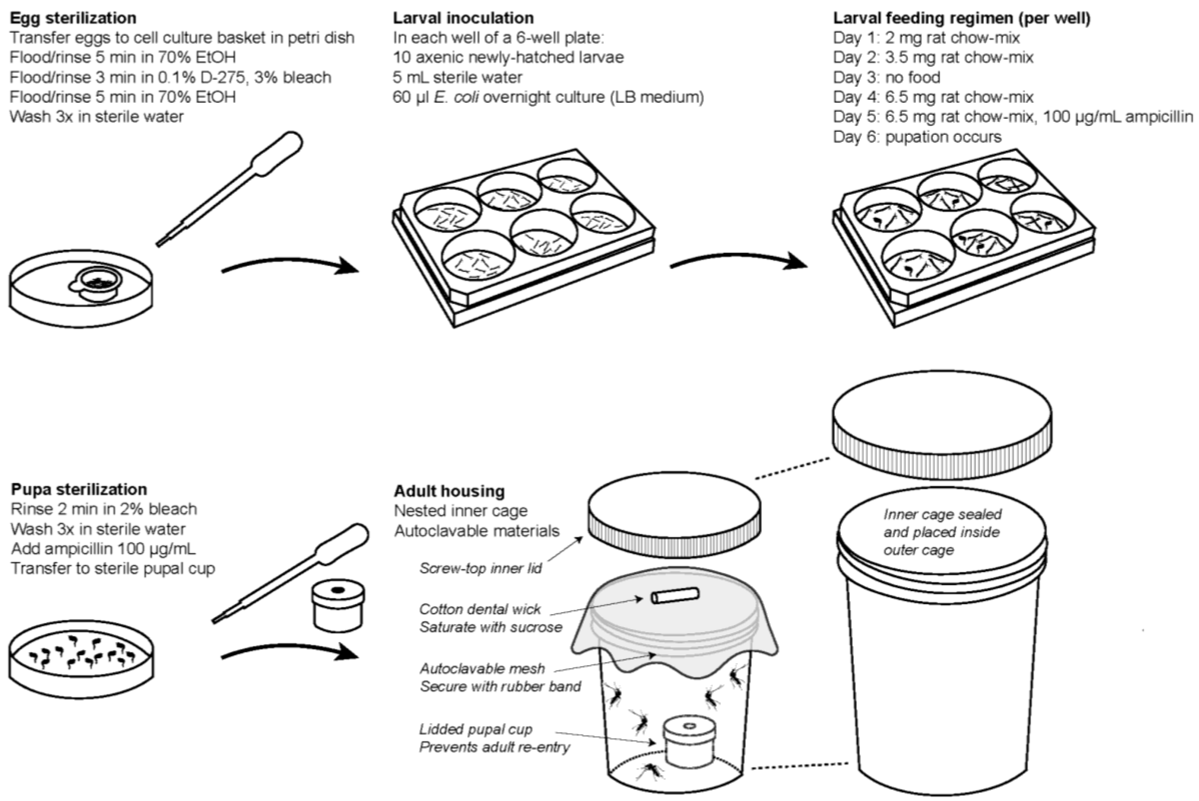

Supplementary Fig. S1 Rearing protocol to produce AX and CN adults. For AX adults produced by clearance of *E. coli*, AX first instars from surface sterilized eggs were placed in wells of 6-well culture plates containing sterile water and *E. coli* K12 MG1655. Larvae and *E. coli* were fed rat chow mix (RCM) diet. Two days after molting to the fourth instar (day 6), ampicillin was added to culture wells. Most fourth instars molted to the pupal stage which was surface sterilized with 2% bleach and transferred to dishes containing sterile water. Resulting AX adults emerged and were maintained in sterile cages. AX adults produced by feeding larvae H+LA or H+Ec (see Methods) hatched from surface sterilized eggs that were also transferred to 6-well culture plates where they pupated. Pupae were transferred to containers with sterile water from which AX adults eclosed and were maintained in sterile containers. CN (non-sterile) adults were produced from surface sterilized eggs that that were placed in culture wells of 6-well culture plates containing sterile water and microbes from larval rearing pans of our general culture. Resulting CN larvae were fed RCM diet. Pupae were transferred to containers with water from which CN adults

eclosed that were maintained in the same type of containers as AX adults. Although unnecessary, larvae in GN cultures containing *E. coli* K12 and larvae in CN cultures were maintained at 27° C in darkness like AX larvae fed H+LA or H+Ec. Adults for each treatment were also maintained at 27° C with a 12 h light: 12 h dark photoperiod.

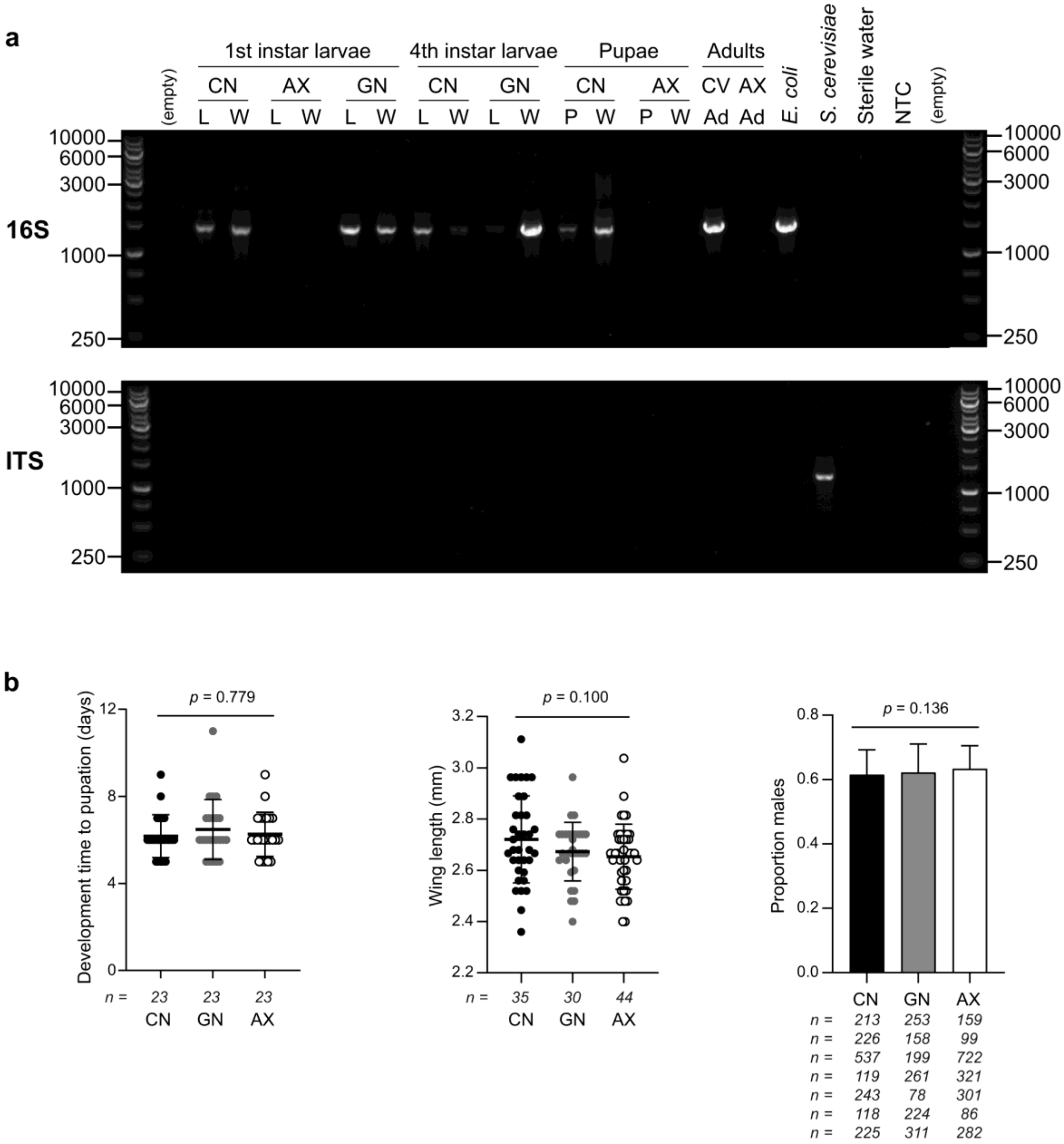

Supplementary Fig. S2 Monitoring for sterility and fitness measures of AX adults produced by clearance of *E. coli* K12. a Each generation of larval, pupal and adult stage mosquitoes were monitored for the presence of bacteria and fungi by endpoint PCR using (top) universal 16S rRNA primers to detect bacteria or ITS primers to detect fungal DNA. L: larva; W: water sample from tissue culture plate well; P: pupa; Ad: adult. Positive controls were DNA extracts from the bacterium *E. coli* or yeast *S. cerevisiae* while negative controls were DNA extracts of sterile water

or a PCR reaction containing all components except template (NTC). For conventional (CN) cultures, bacterial DNA amplicons were always detected in culture water (W) and larvae (L first instars, L fourth instars), pupae (P), and adults (Ad). For AX cultures, no bacterial or fungal DNA amplicons are detected in culture water or larvae (first instars) that hatched from eggs that were surface sterilized. AX larvae do not develop beyond the first instar if fed only RCM diet. AX larvae placed in GN cultures containing *E. coli* K12 and fed RCM diet. Bacterial DNA amplicons are detected in culture water and GN larvae. Addition of ampicillin to fourth instar GN cultures results in clearance of *E. coli* K12 and formation of AX pupae and adults as evidenced by detection of no bacterial or fungal DNA amplicons. The same assays were also used to monitor for sterility of AX adults produced by axenic rearing of larvae fed H+LA medium. AX, GN and CN mosquitoes were additionally monitored by homogenizing individual larvae or adults and plating onto Luria Broth (LB) agar plates which never detected microbes if PCR assays also failed to detect bacterial or fungal amplicons. **b** Development time to pupation (left graph), adult size as measured by wing length (middle graph), and sex ratio of adults (proportion males) (right graph) that emerge from CN cultures, GN cultures reared with *E. coli* to adulthood or GN cultures treated with ampicillin which produces AX adults by clearance. In (**b**), data for development time to pupation (left graph), adult size as estimated by wing length (middle graph) and sex ratio of AX adults produced by clearance of *E. coli* from GN cultures. The left and middle graphs show means ± SD with exact number of individuals measured for each treatment indicated below the x axis. Statistical significance was determined after assessment of homogeneity of variances by a Kruskal-Wallis (development time) test or 1-way ANOVA (adult size). The right graph shows proportion of males that emerged from 7 independent replicates of producing AX adults by clearance of *E. coli* with exact number of individuals per replicate indicated below the x axis. Statistical significance was determined by contingency table analysis. Exact *p* values are indicated in each graph.

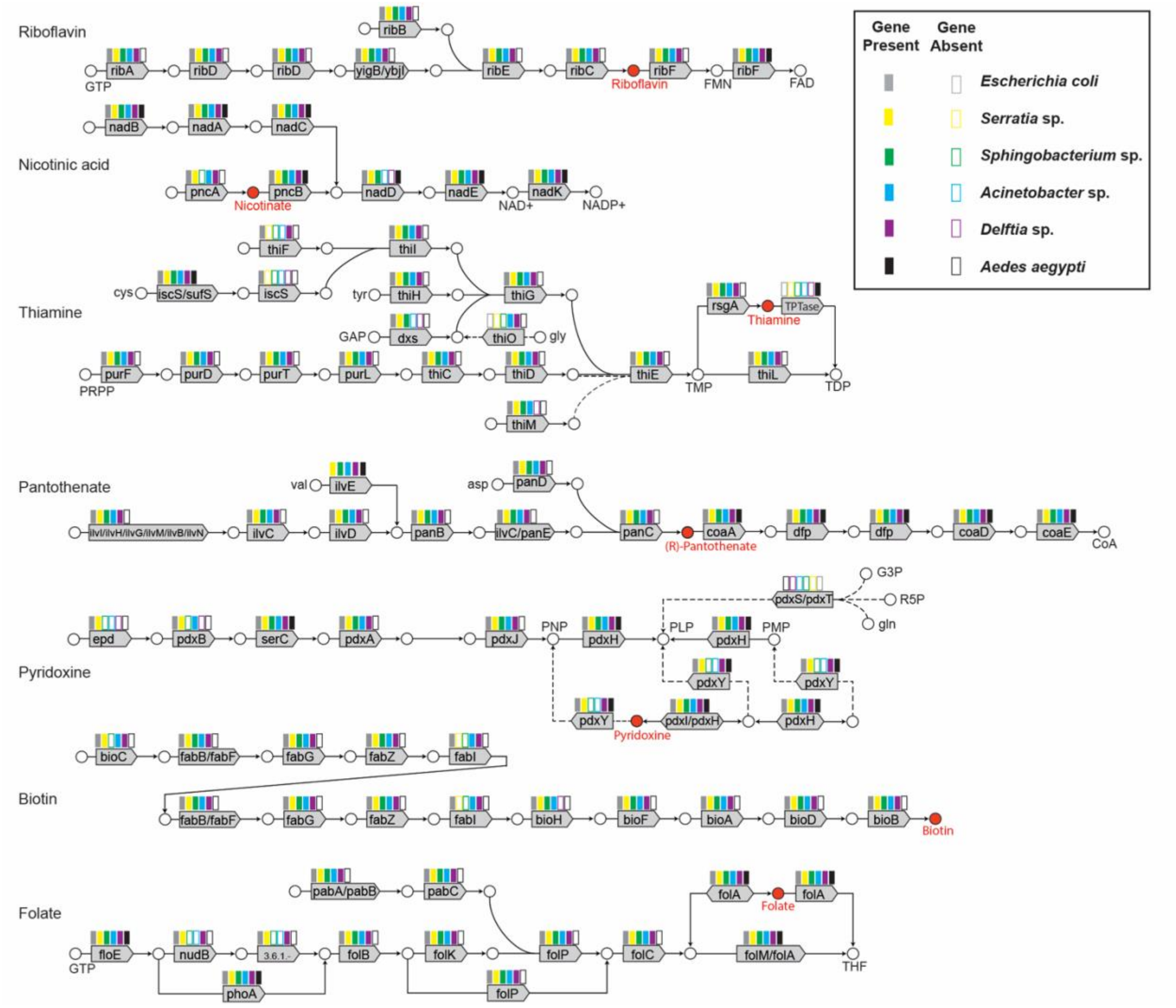

Supplementary Fig. S3 B vitamin and cofactor biosynthesis network reconstruction for *E. coli* K12 (gray box), *Serratia* sp. (UGAL515B_01) (yellow box), *Sphingobacterium* sp. (UGAL515B_02) (green box), *Acinetobacter* sp. (UGAL515B_03) (blue box), *Delftia* sp. (UGAL515B_04) (purple box) and *A. aegypti* (black box). Solid lines denote the primary *de novo* biosynthesis pathways. Dashed lines indicate alternative salvage pathways. Red dots indicate biosynthesized vitamins, open circles indicate major intermediates and cofactors and large grey arrows identify major gene products. Solid boxes above each gene product in a given pathway indicates a homolog was identified in the genome of each species that was examined

while an open box indicates a recognizable homolog was not identified. Abbreviations: Guanosine 5'-Triphosphate (GTP), riboflavin 5'-phosphate (FMN), flavin adenine dinucleotide (FAD), 5-Phosphoribosyl-1-Pyrophosphate (PRPP), Thiamine phosphate (TMP), Thiamine diphosphate (TDP), Cysteine (cys), L-Tyrosine (tyr), D-glyceraldehyde 3-phosphate (GAP), L-Aspartate (asp), L-Valine (val), pyridoxal 5'-phosphate (PLP), pyridoxine 5′-phosphate (PNP), Pyridoxamine 5'-phosphate (PMP).

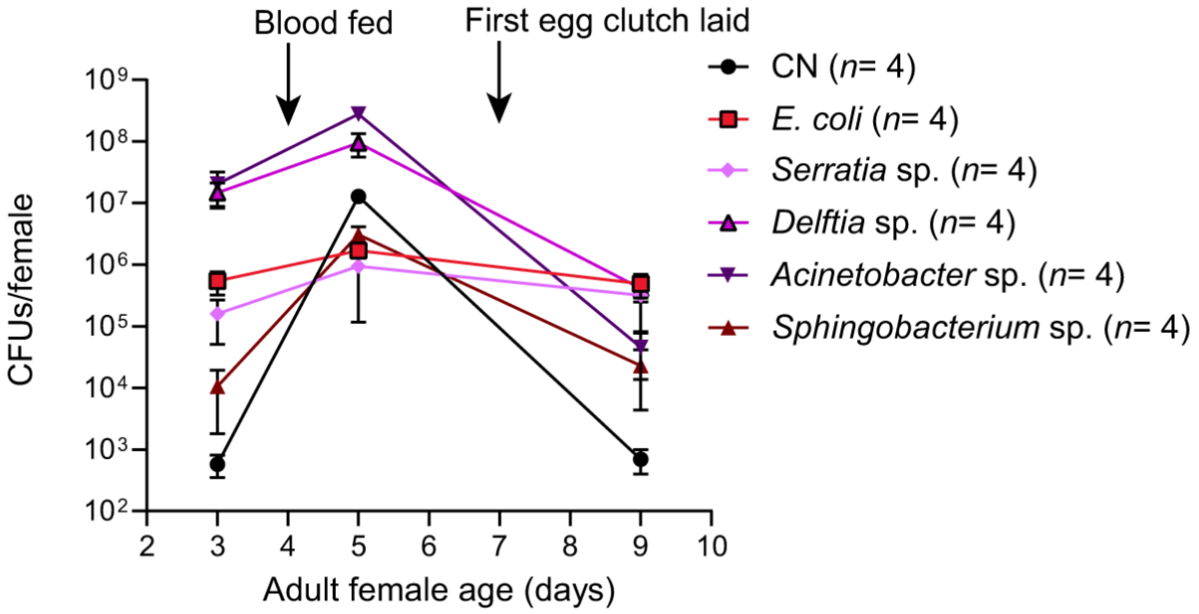

Supplementary Fig. S4 Colony forming units (CFUs) in the guts of females after introducing bacteria in sugar meals to AX females produced by clearance. Mean CFUs per female ± standard error was measured 3, 5, and 9 days old with females blood fed on day 4. The number (n) of replicates measured for each treatment and time point is indicated in the legend with each replicate starting with 10 adult females that were inoculated with each bacterium three days after emerging from the pupal stage. Mortality was higher in adults hosting *E. coli* K12, *Delftia* sp. and *Acinetobacter* sp. after blood feeding and laying eggs but sufficient numbers of females remained alive to measure CFUs on day 9.

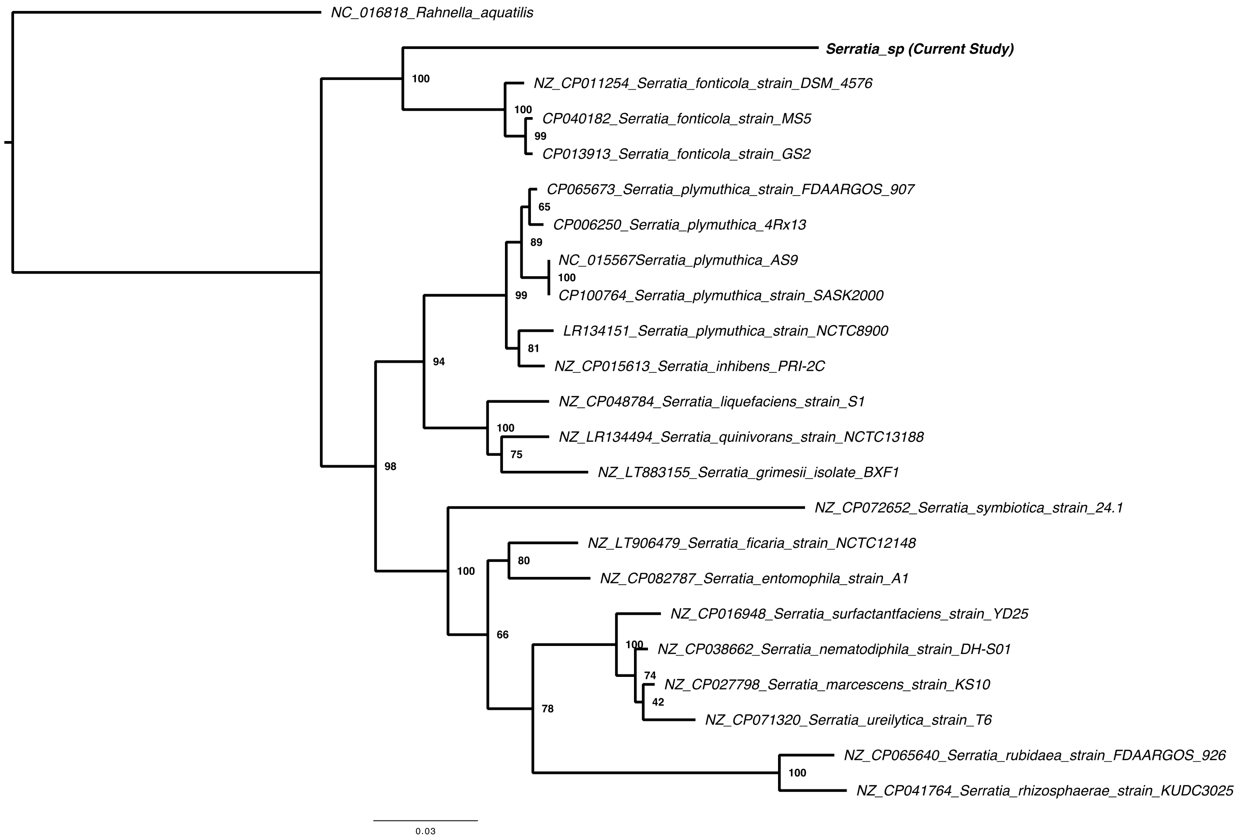

Supplementary Fig. S5 Maximum likelihood tree showing placement of *Serratia* sp. UGAL515B_01 in relation to *S. marcescens* and other members of the genus.

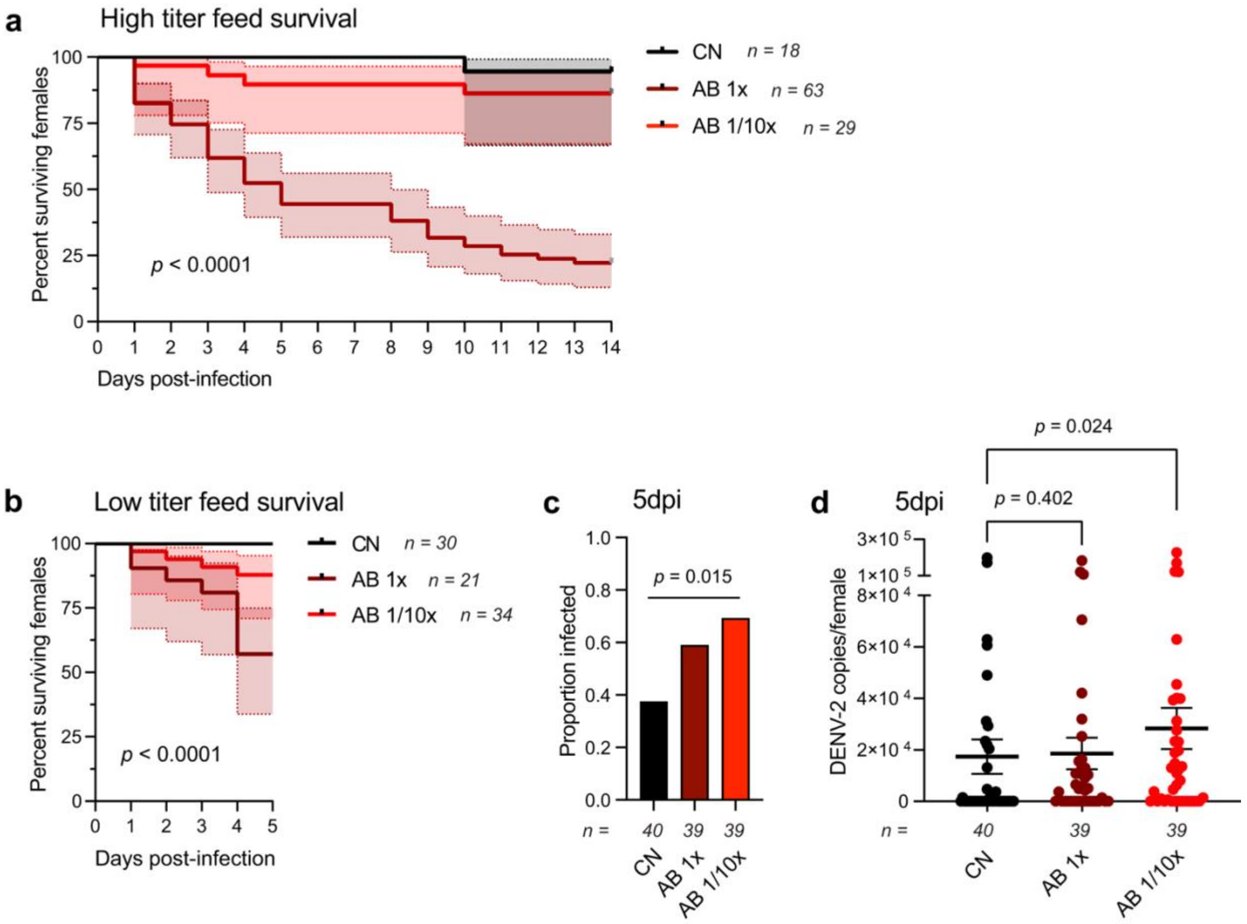

Supplementary Fig. S6 Pretreatment of CN adults with a broad-spectrum antibiotic cocktail dose-dependently increases mortality while increasing DENV-2 infection. a Kaplan-Meier plot showing survival of untreated CN females (no antibiotics) or CN females pretreated with a 1x concentration of antibiotics or a 1/10x concentration of antibiotics (see Methods). Females were fed an infectious blood meal containing DENV-2 at 10^6 $TCID_{50}$/mL and maintained for 14 days with the proportion of surviving adults recorded. **b** Kaplan-Meier plot showing survival of untreated CN females (no antibiotics) or CN females pretreated with a 1x concentration of antibiotics or a 1/10x concentration of antibiotics. Females were fed an infectious blood meal containing DENV-2 at 10^4 $TCID_{50}$/mL with the proportion of surviving adults recorded. **c** Proportion of females infected with DENV-2 at 5 days post-infection (5 dpi) when pretreated with 1x AB, 1/10 AB, or no antibiotics followed by feeding an infectious blood meal containing

DENV-2 at a low titer (10^4 $TCID_{50}$/mL). **d** DENV-2 genome copy number in the midgut of infected females at 5 dpi. In (**a**, **b**), data show survival proportions ± 95% confidence intervals with starting numbers of adult females for each treatment indicated in the figures. Statistical significance was determined by log-rank (Mantel-Cox) tests which indicated survival curves significantly differed among treatments in each graph due to elevated mortality of females treated with AB 1x. In (**c**), data show the proportion of infected individuals is shown with total number of adult females assayed for each treatment indicated below the x axis. Statistical significance was determined by contingency table analysis. In (**d**), data show mean ± SD with exact number of individuals analyzed per treatment indicated below the x axis. Statistical significance was determined after assessment of homogeneity of variances by a Kruskal-Wallis test followed by a post-hoc Dunn's test. Exact *p* values are indicated in each panel of the figure.

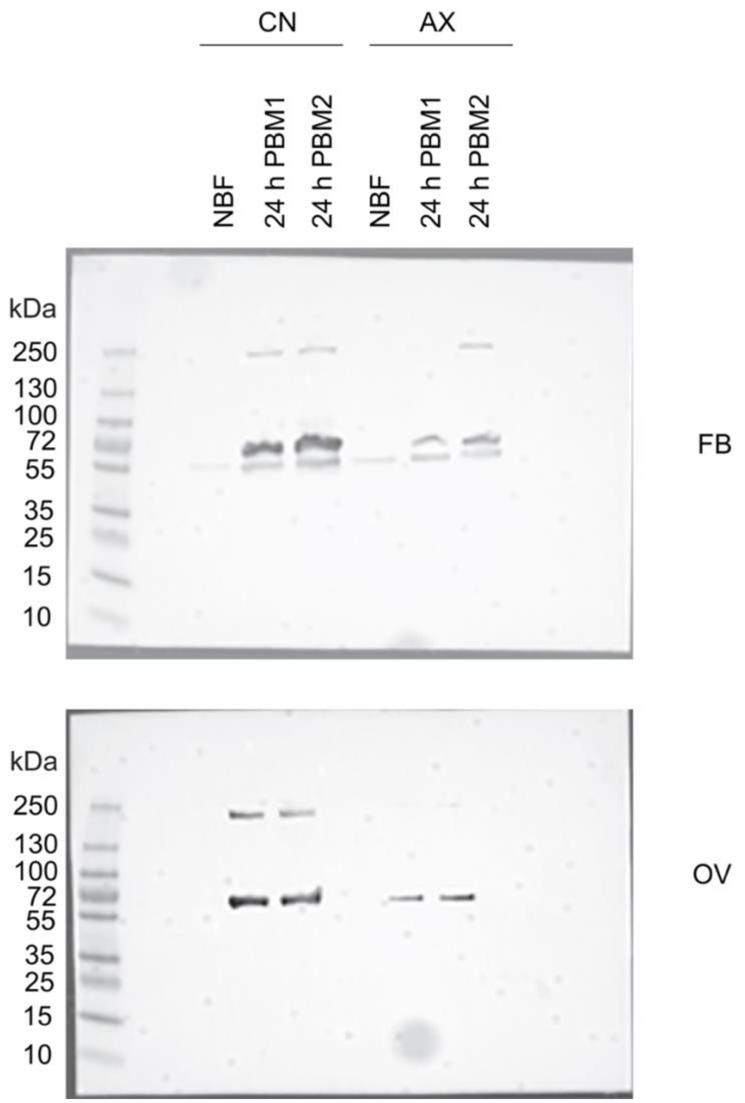

Supplementary Fig S7 Uncropped and unedited immunoblots used for Fig. 2e. The upper blot shows vitellogenin in the fat body (FB) while the lower blot shows vitellogenin in the ovaries (OV) from CN and AX females produced by clearance. Immunoblotting conditions and sample collection from non-blood fed females (NBF), and females 24 h post-blood meal for the first (PBM1) and second (PBM2) gonadotrophic cycle are defined in the legend for Fig. 2e. For each blot, size markers ranging from 250 to 10 kilodaltons (kDa) were loaded in the left-most lane. Vitellogenin (upper bands, ~250 kDa) is processed into the smaller subunit(s) (lower bands, ~55 kDa) shown in Fig. 2e.
