# Supplementary material for: The mosquito Aedes aegypti requires a gut microbiota for normal fecundity, longevity and vector competence
Source: Commun Biol. 2023 Nov 13;6:1154. doi: 10.1038/s42003-023-05545-z (PMC10643675; doi:10.1038/s42003-023-05545-z)
Supplement: Supplementary file 2 — Reporting Summary [file 42003_2023_5545_MOESM2_ESM.pdf]

nature portfolio

Corresponding author(s): Michael R. Strand

Last updated by author(s): 2023-29-10

Reporting Summary

Nature Portfolio wishes to improve the reproducibility of the work that we publish. This form provides structure for consistency and transparency in reporting. For further information on Nature Portfolio policies, see our Editorial Policies and the Editorial Policy Checklist.

Statistics

For all statistical analyses, confirm that the following items are present in the figure legend, table legend, main text, or Methods section.

n/a	Confirmed	
☐	☒	The exact sample size (*n*) for each experimental group/condition, given as a discrete number and unit of measurement
☐	☒	A statement on whether measurements were taken from distinct samples or whether the same sample was measured repeatedly
☐	☒	The statistical test(s) used AND whether they are one- or two-sided<br>*Only common tests should be described solely by name; describe more complex techniques in the Methods section.*
☐	☒	A description of all covariates tested
☐	☒	A description of any assumptions or corrections, such as tests of normality and adjustment for multiple comparisons
☐	☒	A full description of the statistical parameters including central tendency (e.g. means) or other basic estimates (e.g. regression coefficient) AND variation (e.g. standard deviation) or associated estimates of uncertainty (e.g. confidence intervals)
☐	☒	For null hypothesis testing, the test statistic (e.g. *F*, *t*, *r*) with confidence intervals, effect sizes, degrees of freedom and *P* value noted<br>*Give P values as exact values whenever suitable.*
☒	☐	For Bayesian analysis, information on the choice of priors and Markov chain Monte Carlo settings
☒	☐	For hierarchical and complex designs, identification of the appropriate level for tests and full reporting of outcomes
☒	☐	Estimates of effect sizes (e.g. Cohen's *d*, Pearson's *r*), indicating how they were calculated

Our web collection on statistics for biologists contains articles on many of the points above.

Software and code

Policy information about availability of computer code

Data collection	Assembly of bacterial genomes, RNA annotation, and metabolic pathway analyses were conducted using publicly available software as reported in the Methods of the manuscript with citations reported in the references.
Data analysis	Results analyzed using R or Graphpad Prism.

For manuscripts utilizing custom algorithms or software that are central to the research but not yet described in published literature, software must be made available to editors and reviewers. We strongly encourage code deposition in a community repository (e.g. GitHub). See the Nature Portfolio guidelines for submitting code & software for further information.

Data

Policy information about availability of data

All manuscripts must include a data availability statement. This statement should provide the following information, where applicable:

- Accession codes, unique identifiers, or web links for publicly available datasets
- A description of any restrictions on data availability
- For clinical datasets or third party data, please ensure that the statement adheres to our policy

The genomes for the Serratia sp. (strain UGAL515B_01, accession number CP109900), Spingobacterium sp. (strain UGAL515B_02, accession number CP109907), Acinetobacter sp. (strain UGAL515B_03, accession number CP109902), and Delftia sp. (strain UGAL515B_04, accession number CP109906) are submitted to GenBank. All source data used in graphs have been deposited in Figshare.

Research involving human participants, their data, or biological material

Policy information about studies with human participants or human data. See also policy information about sex, gender (identity/presentation), and sexual orientation and race, ethnicity and racism.

Reporting on sex and gender	The research reported in this study did not involve the use of any human participants.
Reporting on race, ethnicity, or other socially relevant groupings	The research reported in this study did not involve the use of any human participants.
Population characteristics	The research reported in this study did not involve the use of any human participants.
Recruitment	The research reported in this study did not involve the use of any human participants.
Ethics oversight	The research reported in this study did not involve the use of any human participants.

Note that full information on the approval of the study protocol must also be provided in the manuscript.

Field-specific reporting

Please select the one below that is the best fit for your research. If you are not sure, read the appropriate sections before making your selection.

☒ Life sciences ☐ Behavioural & social sciences ☐ Ecological, evolutionary & environmental sciences

For a reference copy of the document with all sections, see nature.com/documents/nr-reporting-summary-flat.pdf

Life sciences study design

All studies must disclose on these points even when the disclosure is negative.

Sample size	Sample sizes were chosen on the basis of precedent in the literature or general statistical guidelines that included the use of 30 individuals or more in most assays or a minimum of three independent biological replicates.
Data exclusions	No data were excluded from the results reported.
Replication	All reported results were replicated using independently generated biological samples.
Randomization	Individuals that were analyzed for a given treatment were randomly selected from containers holding many other individauls.
Blinding	Blinding was not possible because all assays required handling of many experimental samples that were generated in a laboratory setting by the authors.

Reporting for specific materials, systems and methods

We require information from authors about some types of materials, experimental systems and methods used in many studies. Here, indicate whether each material, system or method listed is relevant to your study. If you are not sure if a list item applies to your research, read the appropriate section before selecting a response.

Materials & experimental systems

n/a	Involved in the study	
☐	☒	Antibodies
☐	☒	Eukaryotic cell lines
☒	☐	Palaeontology and archaeology
☐	☒	Animals and other organisms
☒	☐	Clinical data
☒	☐	Dual use research of concern
☒	☐	Plants

Methods

n/a	Involved in the study	
☒	☐	ChIP-seq
☒	☐	Flow cytometry
☒	☐	MRI-based neuroimaging

Antibodies

Antibodies used	One in house antibody to A. aegypti vitellogenin was used.

Validation	Validated through prior used published in the literature.

Eukaryotic cell lines

Policy information about cell lines and Sex and Gender in Research

Cell line source(s)	Vero cells. American Type Tissue Collection (ATTC)
Authentication	Authenticated by the source (ATTC)
Mycoplasma contamination	Confirmed to be mycoplasma free by the source.
Commonly misidentified lines (See ICLAC register)	*Name any commonly misidentified cell lines used in the study and provide a rationale for their use.*

Animals and other research organisms

Policy information about studies involving animals; ARRIVE guidelines recommended for reporting animal research, and Sex and Gender in Research

Laboratory animals	No vertebrate animals used in the study. One insect was used: Aedes aegypti UGAL strain.
Wild animals	No wild animals were used int the study.
Reporting on sex	All findings involving blood feeding apply to only one sex of the mosquitoes used in the study (females), because males do not blood feed.
Field-collected samples	No field collected samples were used in the study.
Ethics oversight	The University of Georgia institutional Biosafety Committee provided ethics oversight for the study through approval of safety protocol 2022-0047.

Note that full information on the approval of the study protocol must also be provided in the manuscript.
